# What Are the Determinants of the Sex/Gender Difference in Duration of Work Absence for Musculoskeletal Disorders? A Mixed-Studies Systematic Review

**DOI:** 10.3390/healthcare13243228

**Published:** 2025-12-10

**Authors:** Susan Stock, Nektaria Nicolakakis, Kimberley Cullen, Clermont E. Dionne, Renée-Louise Franche, Valérie Lederer, Joy C. MacDermid, Ellen MacEachen, Karen Messing, Iuliana Nastasia

**Affiliations:** 1 Scientific Group on Work-Related Musculoskeletal Disorders, Department of Environmental and Occupational Health and Toxicology, Quebec Institute of Public Health (INSPQ), Montreal, QC H2P 1E2, Canada; nektaria.nicolakakis@inspq.qc.ca; 2 University of Montreal Hospital Research Centre (CRCHUM), Montreal, QC H2X 0A9, Canada; 3 Department of Social & Preventive Medicine, University of Montreal School of Public Health, Montreal, QC H3N 1X9, Canada; 4 SafetyNet Centre for Occupational Health and Safety Research, School of Human Kinetics & Recreation, Memorial University of Newfoundland, St. John’s, NL A1C 5S7, Canada; kcullen@mun.ca; 5 Laval University Hospital Research Centre (Centre de Recherche du CHU de Québec-Université Laval), Department of Social and Preventive Medicine, Faculty of Medicine, Laval University, Quebec City, QC A1C 5S7, Canada; clermont.dionne@crchudequebec.ulaval.ca; 6 School of Population and Public Health, University of British Columbia, Vancouver, BC V6T 1Z3, Canada; 7 Department of Industrial Relations, University of Quebec in Outaouais (UQO), Gatineau, QC J8X 3X7, Canada; valerie.lederer@uqo.ca; 8 School of Physical Therapy, Western University, London, ON N6G 1H1, Canada; jmacderm@uwo.ca; 9 School of Public Health Sciences, Faculty of Health, University of Waterloo, Waterloo, ON N2L 3G1, Canada; ellen.maceachen@uwaterloo.ca; 10 Interdisciplinary Research Centre on Well-Being, Health, Society and Environment (CINBIOSE), Department of Biological Sciences, University of Quebec in Montreal (UQAM), Montreal, QC H2C 3P8, Canada; messing.karen@uqam.ca; 11 Institut de Recherche Robert-Sauvé en Santé et en Sécurité du Travail (IRSST, Robert-Sauvé Institute of Occupational Health & Safety Research), Montreal, QC H2P 1E2, Canada; iuliana.nastasia@irsst.qc.ca

**Keywords:** musculoskeletal disorders, mixed-studies systematic review, sex, gender, work disability, occupational health

## Abstract

**Background/Objectives:** Musculoskeletal disorders (MSDs) contribute to work disability arising from personal and work-related physical, organizational and psychosocial factors that often differentially affect men and women. We aimed to identify determinants of the sex/gender difference in duration of MSD work absence through a mixed-studies systematic review. **Methods:** We synthesized evidence using the Grading of Recommendations, Assessment, Development and Evaluation (GRADE) approach adapted to prognostic studies and meta-ethnography for qualitative studies, followed by a mixed synthesis. **Results:** Twenty-six quantitative and four qualitative studies contributed to the evidence synthesis. Only two of the twenty-six quantitative studies addressed the sex/gender gap directly, compared to three of the four qualitative studies. Most other quantitative studies provided evidence from sex/gender-stratified analyses of determinants of MSD disability. The synthesis of qualitative studies suggested that domestic strain, less access to modified work/retraining, and gender-biased attitudes of health and insurance system gatekeepers hindered women’s return to work. Prognostic factors in women supporting this conclusion from quantitative studies included the combination “working ≥ 40 h/week and having dependents” and low supervisor support. The mixed synthesis yielded a conceptual model of hypothesized determinants of the sex/gender difference in MSD work disability that integrates factors from personal, workplace, healthcare and insurance–disability management spheres, influenced by the larger sociopolitical, cultural and macroeconomic context. **Conclusions:** Studies directly addressing the sex/gender gap in MSD disability are needed. These can be informed by the proposed model. Practitioners and policymakers can build upon the model to develop and implement MSD prevention and rehabilitation interventions tailored to the needs of men and women to reduce sex/gender disparities.

## 1. Introduction

Musculoskeletal disorders (MSDs) are among the main causes of work disability in industrialized countries, costing billions of dollars annually in direct and indirect expenditures and leading to pain, disability and other human costs [1,2,3,4]. Non-traumatic work-related MSDs (WMSDs) are non-traumatic inflammatory or degenerative disorders affecting musculoskeletal structures of the neck, back and extremities. They arise from cumulative micro-trauma associated with biomechanical and other work exposures, when tissue adaptive and repair capacities are exceeded [5]. Personal risk factors, such as age and previous injury, as well as work-related physical, organizational and psychosocial risk factors, also contribute to the onset of these disorders. The latter are factors related to work organization, management practices, employment conditions and workplace social interactions that increase the probability of harm to worker physical and psychological health [6,7,8]. Examples include high work demands, low decision latitude, low supervisor or coworker support, an effort–reward imbalance, emotionally demanding work and psychological harassment [5,9,10,11,12,13,14]. Organizational and psychosocial work factors may contribute to work-related MSD pathogenesis directly by increasing the intensity or duration of biomechanical exposures. They may also do so indirectly by triggering a stress pathway that leads to increased muscle tension, stress-induced neuroendocrine changes that affect soft tissues and delayed wound healing [15,16,17].

In several jurisdictions over the past several decades, the duration of work absence associated with WMSDs has been steadily increasing among men and women, but it remains higher for women [18,19,20,21,22]. Although determinants of the incidence and duration of prolonged WMSD work disability have been identified [23,24,25,26], these have seldom been examined from a sex and gender perspective. Consequently, the determinants of the sex/gender gap in WMSD work absence duration remain unknown and cannot be addressed in rehabilitation strategies to reduce women’s duration of work absence and promote their return to work (RTW).

Sex refers to the biological attributes that distinguish men and women. Gender refers to the socially constructed and/or socially shaped roles, behaviors, expressions and identities of girls, women, boys, men and gender-diverse people [27]. Gender has a profound impact on health and well-being, including on musculoskeletal health, influencing such behavior and experiences as whether individuals will seek, access and comply with medical help; be exposed to working conditions detrimental to musculoskeletal health; and be responsible for household upkeep and caregiving—which, in turn, can reduce the amount of time for rest and recovery from injury [28,29,30,31]. Sex also impacts musculoskeletal health through a variety of pathways, from interactions with pregnancy and stages of the menstrual cycle, types of muscular development, and interactions with dimensions of work environments and protective equipment to processing of pain and healing [32]. Pathways involving gender often interact with pathways involving sex, making their respective contributions to health outcomes difficult to disentangle, and this is particularly true in occupational health research. For example, differences in MSD work disability between men and women can result from biological differences in anatomy and muscle strength interacting with different work exposures and family constraints related to socially shaped gender roles. Occupational health studies in a real work context (as opposed to laboratory experiments) are seldom able to separate the relative role of biological sex versus societal gender influences observed in these relationships. Hence, the hybrid term “sex/gender” is often used, including in this review, accurately reflecting the frequent interplay of biological and sociocultural influences, and the limits of the literature in this field.

We undertook a mixed-studies systematic review (including both quantitative and qualitative studies) to answer the following research question: “What are the evidence-based determinants of the sex/gender difference in duration of work absence or in the incidence of prolonged work absence for MSDs?” The goal was to identify modifiable factors, including work-related organizational and psychosocial risk factors, that can be acted upon to reduce sex/gender disparities in MSD work disability.

## 2. Methods

The mixed-studies systematic review consisted of the following steps: (1) a bibliographic search; (2) study selection according to inclusion and exclusion criteria; (3) methodologic quality evaluation of retained studies using separate instruments for quantitative and qualitative studies; (4) data extraction and analysis of data from studies of sufficient quality, separately for quantitative and qualitative studies; (5a) synthesis of quantitative studies; (5b) synthesis of qualitative studies; and (6) mixed synthesis integrating the syntheses in 5a and 5b. Results are reported according to the Preferred Reporting Items for Systematic Reviews and Meta-Analyses (PRISMA) [33]. This review was registered with the Open Science Framework a posteriori (https://doi.org/10.17605/OSF.IO/YK9HA (accessed on 7 November 2025)). The review protocol is also available upon request to the authors.

### 2.1. Search Strategy

We searched the 2000–2024 peer-reviewed scientific literature for quantitative and qualitative studies published in English or French. The initial search covered 2000–2017 and 21 bibliographic databases in health and medicine, social sciences, occupational health, business, health policy and women’s studies: Medline (2000–1st week December 2016), Medline In-Process & Other Non-Indexed Citations (2000–30 January 2017), Excerpta Medica Database (EMBASE, 2000–30 January 2017), EBM Reviews/Cochrane Library (includes Cochrane Central Register of Controlled Trials 2000–November 2016, Health Technology Assessment 2000–4th Quarter 2016, NHS Economic Evaluation Database 2000–1st quarter 2015, ACP Journal Club 2000–January 2017, Cochrane Methodology Register 2000–3rd quarter 2012, Cochrane Database of Systematic Reviews 2000–25 January 2017, Database of Abstracts of Reviews of Effects 2000–1st quarter 2015), Cumulative Index to Nursing & Allied Health Literature (CINAHL *with full text*, 2000–31 December 2016), PsycINFO (2000–2017), Psychology & Behavioral Sciences Collection (2000–28 February 2017), SocINDEX (2000–28 February 2017), Ergonomic Abstracts (2000–2017), Canadian Centre for Occupational Health & Safety Databases (HSELINE, CISILO, INRS-Bibliographie, all 2000–2017), Business Source Premier (2000–2017), Women’s Studies International (2000–2017) and the Health Policy Reference Center (2000–2017).

We conducted an updated search covering 1 January 2017 to 3 September 2024 in the three databases that had yielded the most results in the initial search, namely, Medline, EMBASE and CINAHL *complete* (the latter includes approximately twice as many journals as the original CINAHL *with full-text* database). These three databases contributed at least 86% of the results in the original search. The eighteen other databases contributed relatively few articles (six databases contributing no results and five databases contributing five results or fewer, many of the results having also been identified in Medline, Embase or CINAHL).

In addition, we manually searched the reference lists of primary studies meeting the selection criteria (see below) and of literature reviews identified by the search strategy.

The search strategy combined terms from three broad concepts using Boolean logic: “work disability”, “musculoskeletal disorders” and “sex and gender”. Terms pertaining to a fourth concept, “review”, were added to the existing strategy to identify published literature reviews. A sample strategy is given in Supplementary Table S1.

### 2.2. Inclusion and Exclusion Criteria

The titles and abstracts of retrieved records were screened for relevance by one author based on the criteria outlined below and confirmed by a second author. Disagreements were resolved through consensus. We included quantitative and qualitative studies on workers with a musculoskeletal problem that identified explanatory or predictive factors of the duration of MSD work absence, the incidence of prolonged MSD work absence or of another MSD work disability outcome or construct (e.g., time to RTW) and that incorporated a sex- and/or gender-based analysis or a specific gender measure (e.g., identity, role, expression or relations) or that addressed sex/gender issues of a specific sex/gender-based population (e.g., disabled women’s account of the RTW process). We excluded studies (i) that did not include an MSD outcome, (ii) that were cross-sectional quantitative studies, (iii) that had attrition rates ≥ 50%, (iv) that were on workers absent from work for less than two weeks, and (v) that controlled or adjusted analyses for sex/gender without testing for sex/gender interactions or without seeking to explain sex/gender differences in the outcome. We excluded studies with an attrition of ≥50% because we considered that losing at least half the sample to follow-up was a fatal flaw that would make interpretation of reported results very difficult.

### 2.3. Methodologic Quality Appraisal of Selected Individual Studies

Studies meeting selection criteria were independently evaluated by two members of the research team for methodologic quality. Disagreements were resolved through discussion to reach consensus. Studies authored by research team members were not evaluated by these members but by other members of the research team.

The risk of bias in quantitative studies was evaluated as being low, moderate or high with the *Quality in Prognostic Studies* (QUIPS) instrument [34,35]. This instrument allows for studies to be rated for risk of bias in six domains relating to study participation, attrition, prognostic factor and outcome measurement, confounding and statistical analysis, and reporting. Qualitative studies were rated as low-, medium- or high-quality with the *Methodology Checklist: Qualitative Studies* of the National Institute for Health and Care Excellence [36]. This instrument assesses study quality in theoretical approach, design, data collection, validity, analysis and ethics.

A workgroup with expertise on sex- and gender-based research on work-related MSDs from within the study team (in alphabetical order: KC, MK, VL, JM, KM, IN, NN and SS) reflected on 16 criteria for assessing whether sex and gender had been adequately addressed in primary studies based on those of the sex and gender methods appraisal tool developed by co-author JM [37]. Elements proposed by the *Sex & Gender Methods Cochrane Musculoskeletal Group* [38,39] and issues identified by others [32,40,41,42], as well as comments by the study team, also informed work surrounding these criteria, which were discussed, with agreement reached by consensus on their meaning and interpretation. These criteria are listed in Table 1. Three items addressed the research question or conceptual framework of the study, five items addressed the research design, four pertained to the analysis and four addressed the results interpretation. The 16 criteria were integrated in the respective methodologic quality assessment instruments for quantitative and qualitative studies, comprising a seventh domain of bias in the QUIPS instrument and a seventh section in the *Methodology Checklist: Qualitative Studies*.

### 2.4. Data Extraction and Analysis

Data extraction and analysis were carried out only on studies of sufficient quality, that is, on quantitative studies with a low or moderate risk of bias and on qualitative studies of medium or high quality. Information was extracted on author(s), year, country of research, study objective(s), study population (including the proportion of men and women), study design, results and, for quantitative studies, measurement of explanatory or prognostic factors, confounders, and the MSD outcome and effect estimates (e.g., relative risk and hazard ratio) and measures of precision (e.g., 95% confidence interval). The analysis of extracted information was performed separately for quantitative and qualitative studies; it was carried out by one author and confirmed by a second author.

### 2.5. Synthesis of Quantitative Studies

Quantitative studies were grouped by MSD work disability outcome and by explanatory or prognostic factors. For each MSD work disability outcome, the quality of the evidence for an explanatory or prognostic factor based on one or more studies was assessed as being high, moderate, low or very low using the Grading of Recommendations, Assessment, Development and Evaluation (GRADE) approach [43,44,45,46,47,48,49] adapted to prognostic studies [50,51]. These quality ratings reflect the level of confidence in estimated effects across studies and therefore the authors’ confidence in the prognostic evidence.

In the adapted GRADE approach, the initial quality assessment is based on the phase of explanatory prognosis investigation, with phase 1 studies considered initially moderate-quality evidence and phase 2 and phase 3 studies considered initially high-quality evidence, which can be upgraded or downgraded. The phase 1 or exploratory phase of investigation aims to identify associations between potential prognostic factors and an outcome in the absence of prior hypotheses. The phase 2 or confirmatory phase of investigation aims to test or confirm hypothesized associations, while the phase 3 phase of investigation aims to understand the underlying prognostic pathways, such as mediated or moderated effects.

In the GRADE approach, five factors can lead to a decrease in the initial quality rating: (i) study limitations (i.e., risk of bias arising from the design or execution of the studies), (ii) inconsistency in the results across studies, (iii) indirectness (e.g., the population or outcome differs from the one targeted by the research question), (iv) imprecision (e.g., evidence based on a single study or small number of studies, a small sample size for the number of explanatory factors tested, large confidence intervals around results, etc.) and (v) risk of publication bias. Publication bias is possible in prognostic study evidence, but it is less likely when a prognostic factor has been repeatedly addressed, ideally in phase 2 or 3 studies [50]. In the absence of these factors, the initial quality rating can be upgraded if the effect sizes are moderate or large, or there is an exposure–response gradient across studies. Finally, as recommended by the researchers who developed GRADE, we made an overall assessment of the quality of the evidence based on the phase of investigation and the GRADE factors described above, identifying which of these most influenced the assessment and which others contributed to the assessment. But we did not systematically deduct one point for each factor that was not met.

### 2.6. Synthesis of Qualitative Studies

Qualitative studies were synthesized using meta-ethnography [52], an interpretive approach based on inductive reasoning, where concepts and ideas identified in single studies are compared with those in other studies and equivalencies or contradictions sought. This process leads to interpretations that transcend those of the original studies [53,54,55]. Three levels of analysis were conducted. First, concepts and ideas emerging from individual studies were identified (first-order concepts). These were compared across studies to identify those that were recurring or common to more than one study, if possible, given the few qualitative studies of sufficient quality available for analysis. This step required understanding how findings from one study related to those of another study or refuted those of another study; since findings did not refute each other, the process was one of *reciprocal* translation, i.e., translating findings from studies into one another. This gave rise to several second-order concepts. Finally, these second-order concepts were linked together, giving rise to a line of argument expressing the synthesis of findings that was both consistent with results from original studies but also extended beyond them [52,53,54,55].

### 2.7. Mixed Synthesis

As recommended by Sandelowski and colleagues [56,57] and the Joanna Briggs Institute [58], we carried out distinct syntheses of the quantitative and qualitative studies, then proceeded to a mixed synthesis of the two syntheses following a sequential explanatory approach [59]. Concretely, the quantitative synthesis was followed by the qualitative synthesis, and the latter was used to contextualize and make sense of the quantitative findings. The mixed synthesis led to the development of a conceptual model of the hypothesized determinants of the sex/gender difference in the duration of MSD work absence.

## 3. Results

### 3.1. Results of Bibliographic Search

**Figure 1:**
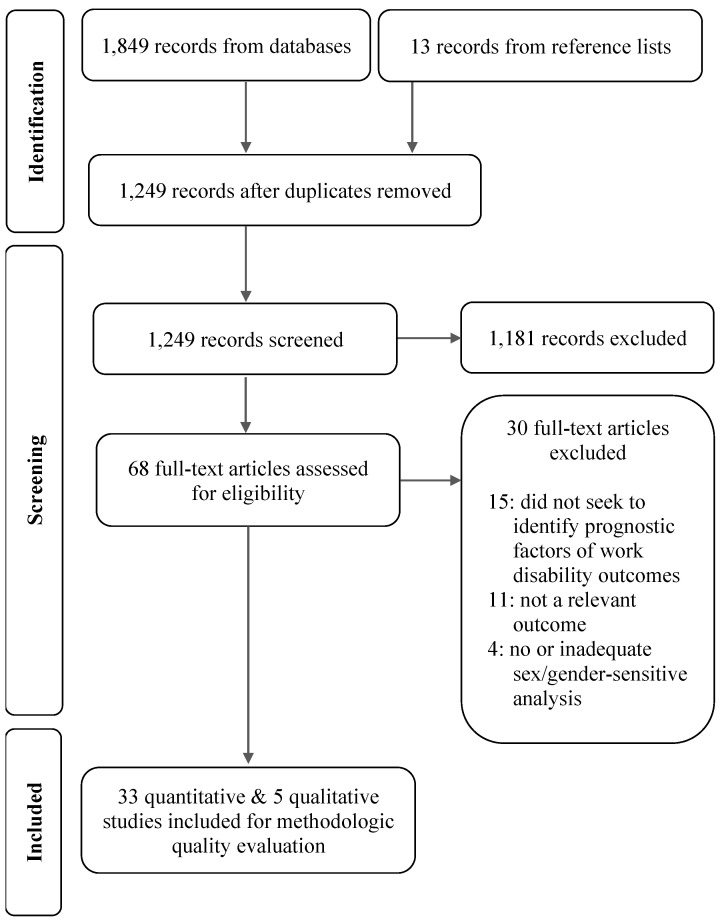
PRISMA flow diagram.

The bibliographic search yielded 1862 records (of which 682 were identified during the updated search). After removal of duplicates and studies that did not meet the selection criteria, 33 quantitative and 5 qualitative studies were included for methodologic quality appraisal (14 of the included quantitative studies were from the updated search). No additional qualitative studies were identified during the updated search. See Figure 1.

### 3.2. Results of Methodologic Quality Appraisal

The methodologic quality assessment of quantitative studies is presented in Supplementary Table S2. This assessment resulted in 7 studies with a low risk of bias [60,61,62,63,64,65,66] and 19 studies with a moderate risk of bias [67,68,69,70,71,72,73,74,75,76,77,78,79,80,81,82,83,84,85] that were retained for analysis, as well as 7 studies with a high risk of bias that were excluded from subsequent analyses [86,87,88,89,90,91,92]. For qualitative studies, the methodologic quality assessment gave rise to four medium-quality studies that were retained for further analysis [93,94,95,96] and one low-quality study that was excluded from further analysis [97]. Details of the methodologic quality assessment of qualitative studies are provided in Supplementary Table S3.

When we considered specifically the results of the evaluation of methodologic quality associated with the treatment of sex/gender, 9 of the 33 quantitative studies (27%) were found to have a high risk of bias. These studies performed particularly poorly with respect to (i) presenting a conceptual or theoretical sex and gender framework; (ii) considering sex and gender at an organizational/system level or at a societal level (e.g., socially constructed roles), in addition to the individual level, during analysis; (iii) testing or considering interactions between sex and gender during analysis; and (iv) failing to discuss the interplay between sex and gender or how results may need to be applied or translated based on sex/gender.

Of the five qualitative studies evaluated, sex and gender were partially addressed in two studies and adequately addressed in three studies. The two studies that partially addressed sex and gender failed to present a theoretical conceptualization of sex and gender, did not consider the interplay between sex and gender during the analysis or interpretation of results, and did not discuss the extent to which study conclusions could be valid across subgroups (for example, different subgroups of women according to marital status and parenthood).

Study design was an area where both types of studies (the nine quantitative and two qualitative studies) performed inadequately with respect to the treatment of sex and gender, particularly when it came to considering different groups of men and women in sampling, inclusion and exclusion criteria (e.g., considering parental or family leave or pregnancy-related preventive leave for occupational health protection reasons provided in certain jurisdictions).

### 3.3. Findings from the Quantitative Literature

#### 3.3.1. MSD Disability Outcomes Examined

In the 26 quantitative studies retained for analysis, four types of MSD disability outcome were investigated: (i) duration of work absence or time in receipt of workers’ compensation benefits (3 studies) [76,77,82], (ii) incidence of or number of episodes of prolonged work absence (5 studies) [62,63,68,74,84], (iii) failure to RTW following prolonged work absence (5 studies) [60,61,67,78,80] and (iv) receipt of social insurance disability pension (13 studies, all from Scandinavian countries) [64,65,66,69,70,71,72,73,75,79,81,83,85].

#### 3.3.2. Sex/Gender Differences in Burden of MSD Work Disability

In 16 of the 26 studies (62% of the studies), the greater burden of MSD work disability occurred in women [62,68,69,70,71,72,73,74,75,79,80,81,82,83,84,85]. In four studies, the burden was greater among men [60,65,66,78]. In three studies [61,63,67], the conclusion about which gender carried the greater burden depended on the specific outcome considered. For example, Dionne et al. [67], assessing a four-category RTW outcome, found that a greater proportion of men failed to return to work at all, whereas a greater proportion of women failed to stay at work after an RTW attempt. Similarly, in Jones et al. [61], women were more likely to return to non-modified duties but also more likely to have a lost-time recurrence after initial RTW. In Pekkala et al. [63], the incidence of prolonged absence was higher among women, but the duration of absence was higher among men. In two other studies [76,77], men’s and women’s durations of work absence were similar. In Siren et al. [64], descriptive data were not available for men and women separately on early exit from paid employment following prolonged absence for shoulder disorder, precluding a conclusion on who bore the greater burden for this outcome. A description of these studies is available in Supplementary Tables S4–S7.

#### 3.3.3. Studies Addressing the MSD Disability Sex/Gender Gap Directly

The GRADE rating of the quality of the evidence for each of the explanatory or prognostic factors examined, grouped by MSD work disability outcome, is presented in Supplementary Tables S8–S11. Only two of the quantitative studies addressed the determinants of the sex/gender gap directly [62,70]. One identified occupation and workplace but with very crude measures that did not specify what aspects of work contributed to this gap [62]. The other [70] identified part-time work as well as several personal factors as determinants of the sex/gender gap in MSD disability: age, income, education, living with children and various MSD diagnoses (rheumatoid arthritis, osteoarthritis, fibromyalgia and neck problems). The remaining quantitative studies provided sex/gender-stratified analyses and identified factors associated with the MSD disability outcome in men, women or both.

#### 3.3.4. Quality of the Evidence for Explanatory Factors of Increased Duration of MSD Work Absence

Supplementary Table S8 presents the quality of the evidence for explanatory factors of increased duration of MSD work absence. Based on a single study [76], there was moderate-quality evidence supporting the following factors in women but not in men: the combination of working at least 40 weekly hours and having dependents; the lack of awareness of a workplace-based occupational health and safety program. Based on the same study, there was moderate-quality evidence in men, but not in women, for the combination of working at least 40 weekly hours and high perceived physical workload and for high job insecurity.

#### 3.3.5. Quality of the Evidence for Explanatory Factors of the Incidence or Number of Episodes of Prolonged MSD Work Absence

With respect to explanatory factors of the incidence or number of episodes of prolonged MSD work absence (Supplementary Table S9), moderate-quality evidence was found that occupation, workplace and occupation–workplace combinations were partially responsible for the female excess in the number of MSD-related work absence episodes of more than two weeks, based on a single study [62]. However, as has been mentioned and as the authors acknowledged, specific working conditions that could account for this excess were not examined. Moderate-quality evidence was found that heavy physical work (involving a lot of walking and lifting) increases the risk of having at least one long-term sickness absence episode compared to sedentary work, in both genders, the effect being strongest among men [68]. Moderate-quality evidence was also found for the following work exposures associated with the MSD disability outcome only in men or only in women: low supervisor support increasing the risk of prolonged work absence (more than eight consecutive weeks) among women, but not men, based on a single study [68]. Based on the same study, there was evidence in men, but not in women, for low job control and for shift work, night work or rotating hours. With respect to personal factors, there was moderate-quality evidence in both women and men for lower parental educational attainment associated with a first episode of work absence greater than 16 days, based on a single study [74]. The effect was stronger in men. In both genders, it was partly mediated through own educational attainment and to a lesser extent by own income or family pattern. There was also moderate-quality evidence for a relationship between weight loss in normal-weight women and an increased rate of prolonged absence episodes; weight gain among normal-weight men was associated with a reduced rate of such episodes [84].

#### 3.3.6. Quality of the Evidence for Explanatory Factors of Failure to RTW

The quality of the evidence for explanatory factors of failure to RTW following prolonged work absence for MSDs is shown in Supplementary Table S10. Moderate-quality evidence was found for the following factors in either women or men. In women, based on a single study [67], factors included increasing job seniority, not being unionized, persistent back pain episode, pain radiating to the upper or lower limbs, fear-avoidance beliefs about activity, and “feeling that the doctor listened carefully while you described your back problem” (a counterintuitive result perhaps reflecting the perceived acknowledgement of the seriousness of the problem).

In men, but not women, based on the same study, the factors associated with failure to RTW were likelihood of losing one’s job in the next two years, perception that job is below one’s qualifications (i.e., underemployment), being on modified duties at consultation, not currently receiving worker’s compensation benefits, dissatisfaction with health services received since consultation (based on focus group results, the study authors hypothesized that this dissatisfaction could serve as an RTW incentive if the patient feels they will not obtain the help they sought), younger age, smoking, poor self-reported health status, higher baseline pain level, at least one previous back pain surgery, and thoracic or cervico-thoracic pain site. Pre-existing anxiety was also a prognostic factor of lower likelihood to RTW in men (its association with RTW was weaker or not statistically significant in women), based on two studies with overlapping samples [60,61], counted as a single study for the purposes of grading the quality of the evidence.

#### 3.3.7. Quality of the Evidence for Explanatory Factors of Receiving an MSD Disability Pension

Supplementary Table S11 presents the quality of the evidence on determinants of receiving a disability pension for an MSD. There was moderate-quality evidence that lower education increases the risk of disability pension in both genders, but this relationship was mediated primarily by working conditions in men and by an equal contribution of working conditions, occupational class and lifestyle variables in women (smoking, alcohol consumption and exercise). Other factors associated with the outcome in both genders for which there was moderate-quality evidence included participation in vocational rehabilitation [64], heavy physical work and working in a forward-bent posture [65,73], and high job demands and low job control. In men and women, the quality of the evidence was low that being overweight or having a common mental disorder increased the risk of disability pension, based on a single study [83].

In women, there was moderate-quality evidence that working in the private sector [64] and that monotonous (repetitive) work increases the risk of disability pension [65]. Conversely, in men, but not women, there was moderate-quality evidence that active jobs (high demands/high control) and passive jobs (low demands/low control) increase the risk of disability pension (the risk was lower for women in active jobs) [65].

Finally, in Lalluka et al. [75], only women were analyzed (there were too few men), and moderate-quality evidence was found that economic difficulties in paying bills and in buying food and clothes increased the risk of a receiving a permanent disability pension for a diagnosed MSD.

### 3.4. Findings from the Qualitative Literature

The four retained qualitative studies examined factors influencing RTW or promoting stay at work for workers with MSDs in Sweden or Norway and are described in Supplementary Table S12. Two studies were focused on young blue-collar men and women, married or cohabiting with children, most of whom had completed vocational rehabilitation [93] or were on part-time disability [96], and one was on men and women outpatients undergoing a vocational rehabilitation program [95]. The fourth study was on women with fibromyalgia who had either stopped working because of their fibromyalgia or were working full-time or part-time [94].

Table 2 presents the first-order concepts associated with sex/gender differences in factors influencing staying at work or returning to work for women and men with MSDs identified in each of the studies. It also presents seven second-order concepts that emerged from the reciprocal translation of studies and identifies the studies associated with each concept: (1) domestic responsibilities; (2) social support; (3) working conditions; (4) gender-biased attitudes and behaviors of gatekeepers; (5) work role and identity; (6) gender differences in injured workers’ expectations, attitudes or beliefs; and (7) transportation to work. The first five of these concepts emerged from at least two of the four qualitative studies, while the last two concepts emerged from single studies. These second-order concepts and how they relate to each other and to staying at work or returning to work are presented below.

#### 3.4.1. Work Role and Identity

The concept of *work role and identity* emerged from three studies. It refers to the idea that work is important for both men and women, not only as a source of income, but also because it contributes to self-esteem and identity, making it a facilitator for RTW.

#### 3.4.2. Domestic Responsibilities

The second-order concept *domestic responsibilities* was apparent in all studies (Table 2), reflecting that most often women shouldered most of the burden of domestic tasks and family responsibilities (e.g., managing household members’ schedules), and, contrary to the men interviewed, they often reported a lack of socio-emotional support from their partners and families. This led to a situation where the needs of the family were prioritized over the women’s own needs—termed *domestic strain* in one study [96]—making it difficult for women to recover, participate in rehabilitation activities, and stay at work or return to work. Conversely, the presence of socio-emotional and practical support from the spouse or family (e.g., sharing domestic work) was a factor that facilitated RTW or staying at work.

#### 3.4.3. Social Support

*Social support* was a second-order concept arising from three studies, which included the idea of socio-emotional support from the partner and family (Table 2) but also manifested itself in other ways. Some of the women with fibromyalgia reported that financial support from their spouse allowed them to reduce their work hours, and this permitted them to continue working. The social contacts and social network provided by work was cited by some of these women as a factor that promoted remaining in a work role or returning to work, whereas women who had stopped working reported a loss of their social network, leading them to feel more isolated. The same was reported by those still working, who, because they used their free time for rehabilitation activities, had a reduced social network outside work. Some women were victims of domestic violence and reported avoiding work to hide bruises and injuries, although others facing domestic violence felt that returning to work could be a better choice because they felt safer at work.

#### 3.4.4. Working Conditions

*Working conditions* was another second-order concept that emerged from two studies, including aspects of work such as physical work demands, organizational restructuring, the work social environment and access to modified work tasks (Table 2). Having a job with high physical work demands hindered returning to work or staying at work for women. In the study on women with fibromyalgia, a context of restructuring of work organization had increased physical work demands, awkward static postures and work pace, which made work harder to execute by those with pain. In addition, this could disrupt social relations and reduce social support from previous coworkers or supervisors. Moreover, there was little opportunity to reduce physical work demands by asking for help from coworkers or having access to modified work because coworkers were also overloaded and many workers with MSDs competed for the relatively few lighter jobs. The ensuing tension and decreased coworker support contributed to a work social environment that tended to negatively impact the decision to return to work or stay at work.

#### 3.4.5. Transportation to Work

Based on the same study of women with fibromyalgia, the second-order concept of *transportation to work* was identified as an obstacle to staying at work or returning to work either because the commute on public transport was very time-consuming (adding up to three hours a day), the use of a personal car increased expenses, or getting to work meant relying on others for lifts when pain made it impossible to drive or use public transportation.

#### 3.4.6. Gender-Biased Attitudes and Behaviors of Gatekeepers

From three studies, the second-order concept *gender-biased attitudes and behaviors of gatekeepers* emerged as an obstacle to women’s RTW (Table 2). The nature of women’s physical work, highly repetitive but low load (perceived as “light work”), often led doctors and social insurance officers to doubt the work-relatedness of women’s musculoskeletal symptoms. This in turn led to reduced access to adequate RTW measures for women. In their interactions with doctors, women, who tended to have non-specific symptoms (e.g., shoulder pain), felt that the legitimacy of their symptoms was questioned and that they were perceived as unmotivated to work. Women were more likely to be offered non-specific treatments (e.g., passive physiotherapy treatments and analgesics) and less active rehabilitation (e.g., when treatment failed, doctors advised women to look for other work without being offered vocational rehabilitation). In contrast, men tended to receive specific diagnoses (e.g., carpal tunnel syndrome or herniated disc) and more specific treatments from doctors. In their interactions with vocational rehabilitation officers, women experienced mistrust, their plans were deemed unrealistic and social security officers decided for them, whereas men felt supported in their path to RTW, being perceived as breadwinners and given access to expensive retraining.

#### 3.4.7. Gender Differences in Injured Workers’ Expectations, Attitudes or Beliefs

Finally, *gender differences in injured workers’ expectations, attitudes or beliefs* regarding the cause of their MSDs and their approach to help-seeking may have influenced the treatments and RTW measures offered to women as compared to men by healthcare professionals and social insurance officers, all of whom were men in the single study from which this concept arose [93] (Table 2). Although both genders attributed injuries to work, women were more likely to blame themselves or excuse the employer for not improving working conditions, whereas men were clearer about holding the employer responsible. Men demanded more specific investigations or additional treatments when those offered were ineffective, and they were more insistent in asking for work modifications and retraining. Women were more likely to persist with the ineffective treatments and were less likely to ask for more than what was offered by the doctors and social insurance officers.

#### 3.4.8. Line of Argument: Synthesis of Second-Order Concepts

From the synthesis of second-order concepts, the following line of argument emerged: domestic strain, less access to modified work and retraining, and the gender-biased attitudes of health and insurance system gatekeepers hindered women’s RTW. These are all modifiable factors that can be acted upon to reduce women’s duration of work absence.

### 3.5. Mixed Synthesis Results

**Figure 2:**
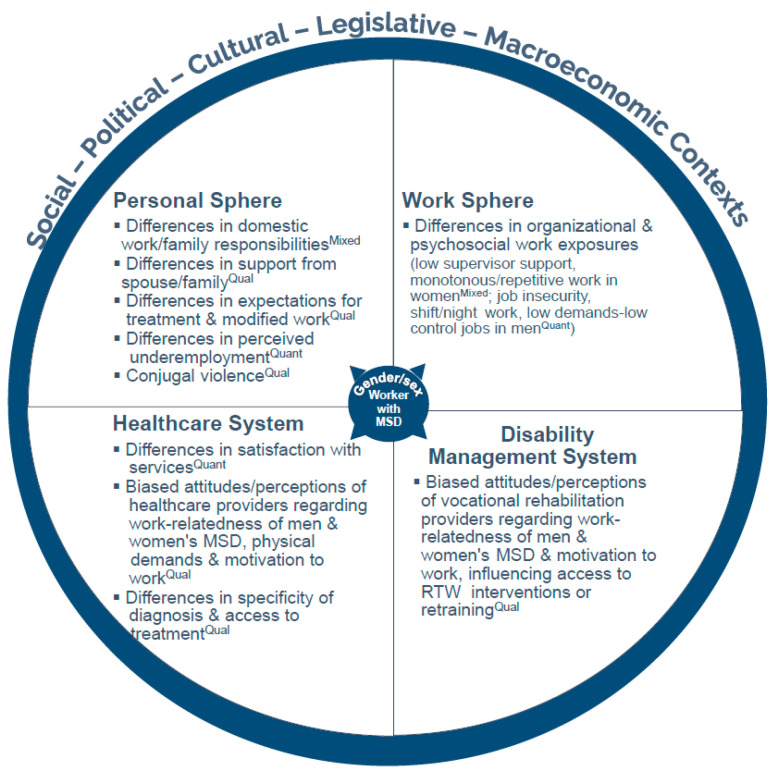
Conceptual model of the hypothesized determinants of the sex/gender difference in musculoskeletal disorder (MSD) work absence duration. RTW: return to work; superscripts in the figure denote determinants emerging from the mixed synthesis (Mixed) or from the quantitative (Quant) or qualitative (Qual) findings.

Using a sequential explanatory approach to synthesize the results of both the qualitative and the quantitative studies allowed us to better understand and interpret some of the quantitative findings. For example, the relevance of some of the prognostic factors identified in quantitative studies for which there was moderate evidence in women, such as the combination “working ≥40 h/week and having dependents” and “low supervisor support” are more easily understood when considering the context provided by the synthesis of the qualitative studies. Based on the four spheres of disability management proposed by Loisel and colleagues [98,99], this mixed synthesis led us to propose a conceptual model of the hypothesized determinants of the sex/gender difference in duration of MSD work absence that integrates factors from the following four spheres, each potentially influenced by the larger sociopolitical, cultural, legislative and/or macroeconomic contexts: (i) the personal sphere; (ii) the work sphere; (iii) the insurance–disability management system; and (iv) the healthcare system (Figure 2).

The model proposed in Figure 2 reflects hypothesized determinants of the sex/gender difference in duration of MSD work disability that emerged from the mixed synthesis, as well as hypothesized determinants from the quantitative and qualitative studies (indicated by the respective superscripts “Mixed”, “Quant” and “Qual” in Figure 2). Findings from the qualitative studies, which addressed the sex/gender gap more directly, were particularly useful to enrich the explanations of how hypothesized determinants could contribute to the sex/gender difference in duration of MSD work disability.

#### 3.5.1. Hypothesized Determinants in the Personal Sphere

In the proposed model, the factors in the personal sphere hypothesized to influence the sex/gender gap in the duration of MSD-related work absence include, among others, inequities in the division of domestic tasks and family responsibilities and women’s tendency to reduce vocational rehabilitation goals to accommodate home responsibilities. This sphere includes emotional and practical support from the spouse or family members, which, when present, is hypothesized to promote women’s RTW or ability to stay at work or, conversely, hinder it when absent. Other elements in this sphere potentially explaining the sex/gender gap in duration of work absence include differences between women and men’s attitudes, beliefs and/or expectations about the work-relatedness of symptoms and their entitlement to receive RTW help, as well as the effects of domestic violence, which, when present, could affect women’s RTW in either direction (i.e., avoiding work to hide bruises or returning to work viewed as a safe environment).

Both biological (sex) and sociocultural (gender) mechanisms may underlie the connections between these determinants and differences in MSD disability between men and women. For example, the factor “inequities in the division of domestic tasks and responsibilities” could lead to longer MSD work disability for women due to the different roles they tend to assume in the home compared to men, such roles likely having been shaped by society (a gender pathway). In turn, these roles could lead to greater exposure of women to physical demands in the home and reduced time for rest and recovery (a gender pathway), interacting with women’s potentially greater susceptibility to muscle fatigue (a biological pathway). There may be other mechanisms at play connecting these determinants to the MSD disability gap, with sociocultural influences acting upstream of biological ones, or vice versa, or biological and sociocultural influences interacting to produce the gap in MSD disability between men and women. However, such mechanisms were rarely addressed in the reviewed literature; indeed, mechanistic studies (phase 3 prognostic studies) were relatively scarce (refer to Supplementary Tables S4–S7, column 1 for the phase of investigation of the analyzed studies).

#### 3.5.2. Hypothesized Determinants in the Work Sphere

Examples in the work sphere hypothesized to explain sex/gender differences in MSD work disability include dimensions of the psychosocial work environment, such as the presence or absence of practical and social support from coworkers or supervisors for women, or of job security for men. Both biological and gender pathways may connect the lack of workplace support to greater MSD disability in women or the lack of job security to greater MSD disability in men, although, as previously mentioned, these pathways were not specifically addressed in the analyzed literature. We may hypothesize, for example, that the lack of practical help at work could directly affect the intensity or duration of biomechanical exposures to which an injured female worker may have to return (gender pathway), potentially interacting with differences in strength (a biological pathway), prolonging her disability, especially if supervisors are unsupportive in accommodating the need for lighter duties because they underestimate her physical demands or the work-relatedness of her musculoskeletal symptoms (a gender pathway). Differences in the nature of men’s and women’s work may also account for a gap in MSD disability (repetitive work emerging as a determinant for women from the mixed synthesis).

#### 3.5.3. Hypothesized Determinants in the Healthcare and Disability Management System Spheres

In the healthcare sphere, we hypothesize that sex/gender differences in MSD work disability and RTW could be influenced by gender-biased attitudes and perceptions of healthcare providers with respect to the work-relatedness of women’s musculoskeletal symptoms and their physical job demands, associated with high repetition but low load work, compared to the high load but less repetitive work of men, and with respect to men’s and women’s motivation to work. The attribution of specific versus non-specific MSD diagnoses, which can lead to less specific or less effective treatments, is also hypothesized to contribute to the sex/gender gap in MSD work disability if the latter diagnoses are more often made in women, as suggested by the qualitative findings.

Similarly, we hypothesize that gender-biased attitudes may partly explain sex/gender differences in MSD work disability if such biases lead vocational rehabilitation providers to be less likely to perceive women’s MSDs as work-related and therefore less likely to offer women retraining or workplace accommodations to facilitate RTW, as suggested by the qualitative findings.

The determinants hypothesized to explain the gap in MSD disability between men and women in the healthcare and disability management spheres more clearly highlight gender mechanisms at play (societal biases), but biological mechanisms may also contribute (for example, stress pathways activated in response to these biases, which can lead to increased muscle tension, stress-induced physiologic changes in soft tissues and delayed wound healing [15,16,17]).

## 4. Discussion

### 4.1. Highlights of Main Findings

Using a mixed-studies approach, we carried out a systematic review of the evidence-based determinants of the sex/gender differences in the duration of work disability associated with MSDs. The results highlight physical, organizational and psychosocial work factors, as well as factors in the personal, healthcare and insurance–disability management spheres that may differently influence men’s and women’s experiences and ultimately their work disability outcomes.

Based on the GRADE assessment of the quality of the evidence, the quantitative studies highlighted some factors specific to women that were predictive of prolonged MSD work absence (e.g., working at least 40 h/week and having dependents, low supervisor support, and fear avoidance about activity), while others factors were specific to men (e.g., the combination of working at least 40 h/week and high physical work demands, shiftwork, fear-avoidance belief about work, being on modified duties for LBP, and currently receiving workers’ compensation for back pain), and other factors were predictive in both men and women (e.g., poor perceived economic status/lower income, lower occupational class, holding multiple jobs, exposure to heavy physical work, working part-time, low worktime control, high-strain work (i.e., high demands combined with low control at work), low expectancy of RTW, and living with children).

This review also highlights gaps in the literature: few quantitative epidemiologic studies specifically sought to identify the factors that contribute to sex/gender differences in MSD work disability. Most studies tended to identify factors associated with the disability outcome in one or both genders, without specifically investigating explanations for the sex/gender gap. Studies investigating sex/gender MSD disability disparities in specific industries or in a broader range of industries or occupations were also generally lacking. In a few studies where industry information was provided, the industry variable was treated as a confounder in multivariate analyses. In the only study with industry-stratified analyses, three broad industry groups were examined (manufacturing, transportation and storage, and health and social work), but analyses could only be carried out in two of the three because of the small number of men or women in the third group [65]. Industry- or occupation-specific studies could shed better light on the relevant modifiable factors according to occupational context. Such studies may provide insight into whether the same factors are relevant for reducing sex/gender disparities in MSD disability across workers in, for example, healthcare, manufacturing, construction or agriculture, or in industries that rely heavily on immigrant or precarious workers. Our proposed model and its four disability spheres could help orient future efforts by providing a basis for new hypotheses and making visible the contribution of some of the lesser-known disability spheres. Given that MSDs are the leading cause of work disability in industrialized countries [2,3,4], such research is urgently needed to reduce the human and economic costs of MSD disability for workers and employers, as well as health and compensation systems and society at large.

### 4.2. Comparison to Other Studies

Many of the personal and work-related variables associated with duration of work absence in the quantitative studies have been found in numerous other studies [23,25,26,98]. However, these studies, like the ones we reviewed, identify prognostic factors of prolonged work disability but do not directly address our research question about the determinants of sex/gender differences in duration of MSD-related work disability. Gender biases of healthcare providers and vocational rehabilitation professionals, described in the four Scandinavian qualitative studies in this review, has also been documented in North American studies [100,101,102,103]. Similarly, the effects on work disability of the large burden of domestic and family responsibilities that falls more heavily on women found in the qualitative studies of this review have also been described in the only other published review we found that meaningfully addressed gender and work disability [104].

### 4.3. Implications of Findings

This review suggests a complex interplay among physical, organizational and psychosocial work factors; personal factors; and system-level factors relating to healthcare and insurance–disability management that influence work disability, often resulting in a greater MSD burden for women. Gendered roles in the personal sphere and potential gendered treatment in the professional sphere and from healthcare providers and disability management professionals may lead to a lack of recognition of the work-relatedness of women’s MSDs and subsequent lack of access to adequate workplace accommodation and retraining. These findings suggest that such factors need to be considered in preventive and rehabilitation interventions aimed at RTW following an MSD.

This has implications for multiple stakeholders in the workplace, in the healthcare system, in rehabilitation settings, and in workers’ compensation and other disability management and insurance systems or social welfare pension programs. Some actionable recommendations to consider are offered below:

- In many countries it is primary care professionals and medical specialists in the community who are the first to evaluate and treat workers in their practices, and they need to be trained to better diagnose and treat WMSDs.
- In relation to employers and relevant occupational health, workers’ compensation and rehabilitation organizations and authorities need to work with healthcare professionals to develop effective communication strategies between healthcare providers and workplaces regarding modified work options that reduce exposure to workplace factors that contribute to duration of work disability.
- Those in the workplace, including employers, supervisors, other managers and worker representatives responsible for health and safety and for the RTW of injured workers, and the occupational health personnel providing services within the workplace, should be trained to recognize MSD risk factors that may be specific to men or women, as well as differences between men and women in factors that influence rehabilitation and RTW, and to develop sex- and gender-sensitive approaches to MSD prevention and rehabilitation in collaboration with all relevant stakeholders.
- Similarly, those working in clinical and vocational rehabilitation settings and in workers’ compensation or other work insurance agencies or social welfare pension programs should be trained to recognize potential differences between men and women in terms of MSD risk factors or factors that influence rehabilitation and RTW following an MSD.
  For all these stakeholders, this training could include how to recognize the health risks associated with the work demands experienced by women, including the effects of highly repetitive work with less obvious physical exertion, and the effects of cognitive and emotional demands, especially in service industries where work involves interactions with patients, clients, students and others, such as healthcare and education, for example [105]. This training could also draw attention to the importance of promoting work–life balance necessary for rest and recovery from injury through workplace practices and policies, and the importance of offering more accommodation and greater flexibility with respect to vocational rehabilitation choices for women with MSDs, as well as men.
- Inequities in the division of domestic work highlighted in this review suggest that healthcare providers and vocational rehabilitation professionals should be attentive to the need to provide support for women with respect to these issues and ensure that they receive resources to address and reduce their domestic burdens to promote recovery and RTW.

These strategies can help these workplace actors and service providers in each of these settings to improve their practices and reduce health disparities between men and women.

### 4.4. Strengths and Limitations of the Review

One of the original methodologic contributions of this study was the integration of 16 criteria for assessing risk of bias associated with the treatment of sex and gender in both quantitative and qualitative studies of explanatory or predictive factors of MSD work disability (presented in Table 1). These criteria can be easily integrated into other existing risk-of-bias instruments to evaluate both quantitative and qualitative predictive or explanatory studies. They may be useful to others undertaking systematic reviews or to those planning and/or reporting on prognostic studies. One of the co-authors of this paper and her colleagues have recently also used these criteria to evaluate the treatment of sex and gender in intervention research [106,107].

Another strength was the breadth of coverage of the bibliographic search of the quantitative and qualitative literature in English and French across 21 bibliographic databases. However, our search did not cover the gray literature, and the updated search for the period 2017–2024 covered 3 of the 21 original databases (albeit it was those three databases that had identified at least 86% of the results in the initial search). It is nonetheless possible that some relevant studies were missed that could reveal other determinants of the sex/gender difference in duration of musculoskeletal work disability or contradict some of the findings reported herein.

The results of this review highlight the limits of existing epidemiologic studies of MSD prognostic factors to adequately take sex and gender into account and the limits of current research design strategies in identifying determinants of sex/gender differences in MSD work disability. As previously mentioned, we found few quantitative epidemiologic studies that directly addressed our research question and specifically identified determinants of the sex/gender gap in duration of MSD work absence. This is an important limitation in the evidence base, and our review reflects this state of the science. In general, the epidemiologic studies included in this review tended to focus on individual-level prognostic factors and rarely considered organizational or societal factors, nor did they consider other factors, such as race, ethnicity, or immigrant or minority status, that can interact with each other, as well as with sex, gender and work exposures, and potentially increase MSD disability risk.

Moreover, variables in the quantitative studies did not adequately capture the issues identified in the qualitative studies. Even when measuring variables related to work exposures, the epidemiologic studies used crude measures that did not capture the differences in intensity, duration or frequency of exposures, or in the nature of these exposures in women versus in men, that might influence MSD work disability (for example, measures only assessing load in a healthcare establishment will fail to capture that women may be lifting people because they are nurses and men lifting objects because they are maintenance workers). Therefore, for many work exposures, the relationship with the work disability outcome was demonstrated in both men and women. This may be due in part to a lack of a sex and gender theoretical framework that would facilitate the formulation of sex/gender-relevant research questions and sex/gender-sensitive instruments and indicators. In the absence of such considerations, sex/gender-stratified analyses, while important, will not be sufficient to address the sex/gender gap in MSD work disability.

Finally, most of the quantitative studies were derived from Scandinavian countries, the rest from Canada, reflecting developed economies with universal healthcare systems and relatively strong social welfare policies. This potentially limits the generalizability of the findings to other contexts, such as the United States or many developing countries where greater barriers may exist in accessing healthcare and occupational rehabilitation services. This is potentially an important limitation of this review. However, even in Canadian and Scandinavian countries, our review highlighted sex/gender differences in MSD work disability that are potentially relevant to other jurisdictions These differences suggest that more work needs to be done to address them even in these countries and that gaps may be even more pronounced in other contexts. It would be informative to carry out similar studies in a wider range of geographic areas with a wider variety of health and compensation system and economic and social policy contexts.

The qualitative studies addressed the sex/gender gap in MSD work disability more directly and generated more detailed results, but their generalizability is unknown or limited; moreover, we found very few qualitative studies, only four of adequate methodologic quality, all Scandinavian, looking at MSD-related disability pension outcomes. Nonetheless, their findings are relevant to the North American context, and, as mentioned above, others have described similar issues in North American studies.

### 4.5. Future Directions

The hypotheses generated in the qualitative studies and presented in the conceptual model should be tested in better-designed quantitative studies that more effectively consider sex and gender at every stage of the research process: formulating research questions that clearly articulate sex or gender issues that are relevant to the population being studied; considering sex and gender in sampling, inclusion and exclusion criteria, and in recruitment; considering exposure, outcome and covariate measures that capture relevant biological and sociocultural influences that impact MSD work disability in men and women and avoiding gender-biased measures of exposure and outcome that skew heavily to only one gender’s experience; articulating how sex and gender will be considered in analyses. Moreover, when relevant, sex and gender should be considered at an individual, organizational, and system and/or societal level. The extent to which the conclusions are valid for both men and women, as well as for gender-diverse populations, should be considered. Finally, future studies should explore links between the social, political, cultural, legislative and macroeconomic contexts and more proximal determinants of sex/gender differences in MSD disability, and whether and how the sex/gender gap is changing over time.

## 5. Conclusions

Studies directly addressing the sex/gender gap in MSD disability are needed. These can be informed by the proposed conceptual model of the determinants of this gap emerging from the personal, work, healthcare and insurance–disability management spheres. This model can serve as a basis for future research and support practitioners and policymakers in developing and implementing more effective MSD prevention and rehabilitation interventions tailored to the needs of men and women, thereby contributing to reducing MSD disability and sex/gender-based disparities in disability outcomes. A greater and more thoughtful integration of sex/gender; other social determinants of health such as race, ethnicity, and immigrant or minority status; or other potentially relevant factors in WMSD research could catalyze new better-quality evidence, which in turn could support more effective population- and organization-level interventions, policies and outcomes.

## Figures and Tables

**Table 1:** Tool to evaluate the treatment of sex and gender in primary research studies.

Research Question/Conceptual Framework	Yes	Partially	No	N/A
The study clearly articulates sex or gender differences/issues that are relevant to the research question or context, e.g., epidemiology, risk factors and differential outcomes.				
The study discusses a theoretical conceptualization of sex/gender.				
The study clearly articulates a sex/gender research question as a primary or secondary research question, or states that the purpose includes controlling for/measuring the effect of sex/gender in the primary research question.				
**Study Design**				
Sex/gender and diverse populations of men and women are considered in sampling, inclusion and exclusion criteria (e.g., including parental leave, family leave or preventive withdrawal related to pregnancy).				
The study describes the recruitment strategies to accrue the appropriate sample of men and women.				
Measures of exposure/covariates are not gender-biased.				
The study describes how sex and gender were measured, and measures were appropriate.				
Choice of outcome measures or diagnostic/validation tests is not gender-biased.				
**Analysis**				
A description of how sex/gender are handled is stated in the data analysis plan (sex-disaggregated or stratified analyses, pathway modeling, treatment of sex and gender variables).				
Sex and gender are considered at an individual, organizational/system and/or societal level (e.g., gender relations and socially constructed roles).				
If applicable, potential interactions/confounding between sex and gender are considered/tested.				
The validity of the results for men and women is tested.				
**Interpretation**				
The extent to which the conclusions are valid for men and women is stated.				
The extent to which the conclusions are valid across gender-diverse populations is stated.				
Potential confounding, interaction and/or interplay between sex and gender are considered.				
How the results may need to be applied/translated based on sex/gender is considered.				
**Overall Assessment: Are sex and gender adequately addressed in the study?**				
Comments				

**Table 2:** Factors influencing return to work or promoting stay at work of workers with musculoskeletal disorders in qualitative studies.

Concepts	Ahlgren and Hammarström, 2000 [93]	Liedberg and Henriksson, 2002 [94] (Study on Women with FM)	Östlund et al. 2004 [96]	Kvam et al. 2013 [95]
**Domestic responsibilities**	x	x	x	x
Inequity in the division of domestic tasks	x	x	x	x
Inequity in the division of family responsibilities	x	x	x	
Socio-emotional and practical support from partner and family		x	x	
**Social support**		x	x	x
Social isolation		x	x	
Social network		x		
Social life		x		x
Socio-emotional and practical support from partner and family		x	x	
Financial support from partner		x		
Domestic violence			x	
**Working conditions**	x	x		
Physical work demands	x	x		
Organizational restructuring		x		
Work social environment		x		
Access to accommodations/modifications of schedules and other working conditions		x		
**Gender-biased attitudes and behaviors of gatekeepers**	x	x		x
Healthcare professionals	x			x
Social insurance/vocational rehabilitation officers	x	x		
**Work role and identity**	x	x		x
Work contributing to self-esteem and identity	x	x		x
Work as a source of income	x	x		
**Gender differences in injured workers’ expectations, attitudes or beliefs**	x			
Attribution of the cause of work injury	x			
Help-seeking approach	x			
**Transportation to work**		x		
Time-consuming commute		x		
Additional expenses incurred by using personal vehicle		x		
Reliance on others for transportation		x		

Second-order concepts are presented in the row headers. First-order concepts appear as bullets under each header. FM: fibromyalgia. x: indicates that the concept emerged from the study.

## Data Availability

No new data were created or analyzed in this study. Data sharing is not applicable to this article.

## Supplementary Materials

The following supporting information can be downloaded at https://www.mdpi.com/article/10.3390/healthcare13243228/s1: Table S1. Sample search strategy in Medline; Table S2. Evaluation of the risk of bias in quantitative studies of explanatory factors of musculoskeletal disorder work disability duration in women and men; Table S3. Methodologic quality evaluation of qualitative studies of factors influencing return to work of women and/or men with musculoskeletal disorders; Table S4. Description of quantitative studies of explanatory factors of the duration of work absence/workers’ compensation (WC) for a musculoskeletal disorder (MSD) in women and men; Table S5. Description of quantitative studies of explanatory factors of episodes of prolonged work absence for musculoskeletal disorders (MSDs) in women and men; Table S6. Description of quantitative studies of explanatory factors of failure to return to work (RTW) after work absence or prolonged wok disability for a musculoskeletal disorder (MSD) in women and men; Table S7. Description of quantitative studies of explanatory factors of receiving a social insurance disability pension (DP) for a musculoskeletal disorder (MSD) in women and men; Table S8. Quality of the evidence on explanatory factors of the duration of work absence/workers’ compensation (WC) for a musculoskeletal disorder (MSD) in women and men; Table S9. Quality of the evidence on explanatory factors of incidence or number of episodes of prolonged work absence for a musculoskeletal disorder (MSD) in women and men; Table S10. Quality of the evidence on explanatory factors of failure to return to work (RTW) following work absence for a musculoskeletal disorder (MSD) in women and men; Table S11. Quality of the evidence on explanatory factors of receiving social insurance disability pension (DP) for a musculoskeletal disorder (MSD) in women and men; Table S12. Description of qualitative studies of factors influencing return to work and/or stay at work of women and men with musculoskeletal disorders (MSDs).
