# Supplementary material for: What Are the Determinants of the Sex/Gender Difference in Duration of Work Absence for Musculoskeletal Disorders? A Mixed-Studies Systematic Review
Source: Healthcare (Basel). 2025 Dec 10;13(24):3228. doi: 10.3390/healthcare13243228 (PMC12732891; doi:10.3390/healthcare13243228)
Supplement: Supplementary file 1 [file healthcare-13-03228-s001.zip › healthcare-3912839-supplementary.pdf]

Supplemental Table S1. Sample search strategy in Medline

Concepts	Search terms
1) Work disability	(((sick* or illness or disability) adj (day* or leave*)) or ((sick* or work or ill*) adj1 absence*) or (absent adj1 (work or sickness)) or ((period* or long-term or longterm or prolonged or longer or shorter or reduced) adj1 (absence* or disabilit*)) or absenteeism or (time adj1 loss) or ((absent or lost) adj (time or workday* or work-day*)) or ((return or back) adj1 (work* or job)) or "back to work" or "work disability" or "wage replacement" or "time on benefit*" or "benefit duration" or (worker* adj2 (compensat* or indemnit*))).ti,ab,kw.<br>OR "workers' compensation"/ or workman compensation/ or sick leave/ or medical leave/ or absenteeism/ or return to work/ or job accommodation/ or work resumption/ or *"workers' compensation"/ or *workman compensation/ or *sick leave/ or *medical leave/ or *absenteeism/ or *return to work/ or *work resumption/
2) Musculoskeletal disorder	((musculoskeletal or musculo-skeletal) adj4 (disorder* or disease* or injur* or pain or symptom*)).ti,ab,kw.<br>OR ((back*1 or neck*1 or limb*1 or extremit* or hand*1 or wrist* or elbow* or shoulder* or arm*) adj4 (disorder* or injur* or pain or symptom*)).ti,ab,kw.<br>OR ("cumulative trauma disorder*" or (repetiti* adj1 (strain or motion) adj1 (disorder* or injur*)) or ((overuse or over-use or overexertion or over-exertion) adj2 syndrome*)).ti,ab,kw.<br>OR ("carpal tunnel syndrome" or epicondylitis or tendonitis or tenosynovitis).ti,ab,kw.<br>OR musculoskeletal diseases/ OR arm injuries/ OR forearm injuries/ OR tennis elbow/ OR wrist injuries/ OR back injuries/ OR spinal injuries/ OR hand injuries/ OR neck injuries/ OR occupational diseases/ OR back pain/ OR low back pain/ OR neck pain/ OR exp "sprains and strains"/ OR exp cumulative trauma disorders/
3) Sex and gender	gender*.ti,ab,kw.<br>OR sex factors/ or sex distribution/ or sex ratio/ or women, working/ or sex difference/ or "gender and sex"/ or gender/
4) Review	(((systematic or scoping or critical or state-of-the-art or evidence) adj2 (review* or overview*)) or (scoping adj (study or studies)) or meta-analys#s or metaanalys#s).ti,ab,kw.<br>OR review literature as topic/ or review/ or meta-analysis as topic/ or meta-analysis/

The search strategy combined terms from concepts 1, 2 and 3. Terms include both natural language and controlled vocabulary terms (Medical Subject Headings) ending with an oblique symbol (/). Terms pertaining to concept 4 were added to the existing strategy to identify published literature reviews. Similar strategies were used for each bibliographic database, adapting controlled vocabulary terms as needed.

Supplemental Table S2. Evaluation of the risk of bias in quantitative studies of explanatory factors of musculoskeletal disorder work disability duration in women and men

Study	Domain of bias							Overall risk of bias
	Study participation	Study attrition	Prognostic factor measurement	Outcome measurement	Study confounding	Statistical analysis & reporting	Treatment of sex & gender	
Jones et al., 2023 [61]	L	L	L	L	M	L	L	L
Jones et al., 2021 [60]	L	L	L	L	M	L	L	L
Laaksonen et al., 2010 [62]	L	L	L	L	M	L	L	L
Pekkala et al., 2018 [63]	L	L	L	L	H	L	M	L
Siren et al., 2019a [65]	L	L	M	L	M	L	M	L
Siren et al., 2019b [64]	L	L	M	L	M	L	M	L
Siren et al., 2020 [66]	L	L	L	L	M	L	M	L
Foss et al., 2011 [68]	H	L	M	L	M	L	L	M
Dionne et al., 2007 [67]	M	M	M	L	L	M	M	M
Gjesdal et al., 2004 [69]	L	L	L	L	H	M	M	M
Gjesdal et al., 2011 [70]	L	L	L	M	H	M	L	M
Hagen et al., 2000 [71]	L	M	M	M	H	L	H	M
Hagen et al., 2006 [72]	L	M	H	M	M	L	H	M
Kristensen et al., 2007 [74]	L	L	L	L	M	L	M	M
Lederer & Rivard, 2014 [77]	L	L	L	L	H	L	L	M
Opsahl et al., 2016 [80]	M	L	M	M	L	L	M	M
Smith et al., 2014 [82]	L	L	M	M	M	M	M	M
Lalluka et al., 2015 [75]	L	M	L	L	M	M	H	M
Lederer et al., 2012 [76]	H	L	M	L	L	L	L	M
Vahtera et al., 2010 [85]	L	L	L	L	M	L	M	M
Maas et al., 2018 [78]	L	L	M	L	M	L	L	M
Svard et al., 2018 [83]	L	L	M	L	M	L	M	M
Svard et al., 2020 [84]	L	M	M	L	M	L	M	M
Salonen et al., 2020 [81]	L	L	M	M	M	M	M-H	M
Kjellberg et al., 2016 [73]	M	L	M	L	M	L	M	M
Mantyniemi et al., 2012 [79]	M	L	L-M	M	M	L-M	M	M
Holte et al., 2000 [89]	L	M	M	L	H	H	H	**H**
Borg et al., 2001 [86]	L	L	L	L	H	H	H	**H**
Gatchel et al., 2005 [87]	H	H	L	M	M	H	H	**H**

Lahelma et al., 2012 [91]	M	L	M	L	H	L	H	**H**
Kaiser et al., 2008 [90]	H	M	M	L	M	H	L	**H**
Gjesdal et al., 2018 [88]	M	M	L	H	H	M	H	**H**
Oliv et al., 2019 [92]	H	L	H	M	M	H	L	**H**
**No. studies rated H**	5	1	2	1	9	5	9	**7**

L: low risk of bias, M: moderate risk of bias, H: high risk of bias

Supplemental Table S3. Methodologic quality evaluation of qualitative studies of factors influencing return to work of women and/or men with musculoskeletal disorders

Study	Section 1: theoretical approach		Section 2: study design	Section 3: data collection	Section 4: validity			Section 5: analysis						Section 6: ethics	Section 7: sex/gender	Overall study quality
	Is a qualitative approach appropriate?	Is the study clear in what it seeks to do?	How defensible/ rigorous is the research design/ methodology?	How well was the data collection carried out?	Is the role of the researcher clearly described?	Is the context clearly described?	Were the methods reliable?	Is the data analysis sufficiently rigorous?	Are the data 'rich'?	Is the analysis reliable?	Are the findings convincing?	Are the findings relevant to the aims of the study?	Are the conclusions adequate?	How clear & coherent is the reporting of ethical considerations?	Were sex & gender adequately addressed in the study?	
Ahlgren & Hammarström, 2000 [93]	Appropriate	Clear	Partially rigorous	Appropriate	Clear	Partially described	Partially reliable	Rigorous	Somewhat rich	Partially reliable	Convincing	Relevant	Partially adequate	Not sure/not reported	Adequately addressed	M
Kvam et al., 2013 [95]	Appropriate	Clear	Partially rigorous	Partially appropriate	Clear	Clearly described	Partially reliable	Rigorous	Somewhat rich	Reliable	Convincing	Relevant	Adequate	Partially clear	Adequately addressed	M
Liedberg & Henriksson, 2002 [94]	Appropriate	Clear	Rigorous	Appropriate	Unclear	Not sure	Partially reliable	Rigorous	Somewhat rich	Partially reliable	Convincing	Relevant	Partially adequate	Partially clear	Partially addressed	M
Östlund et al., 2004 [96]	Appropriate	Clear	Partially rigorous	Partially appropriate	Clear	Partially described	Reliable	Partially rigorous	Somewhat rich	Reliable	Convincing	Relevant	Adequate	Clear	Adequately addressed	M
Hooftman et al., 2008 [97]	Appropriate	Clear	Partially rigorous	Partially appropriate	Clear	Partially described	Partially reliable	Not rigorous	Somewhat rich	Partially reliable	Not convincing	Relevant	Partially adequate	Clear	Partially addressed	**L**

M: medium-quality study, L: low-quality study

Supplemental Table S4. Description of quantitative studies of explanatory factors of the duration of work absence/workers' compensation (WC) for a musculoskeletal disorder (MSD) in women and men

Study	Study objective(s) (Phase of investigation)	Participants included in MSD-specific analyses	Study design	Work absence outcome(s) measured	Prognostic/ explanatory factor(s) measured	Confounders measured	Results	Study risk of bias
Lederer et al., 2012 [76]	To assess the differential influence of personal & work-related psychosocial, physical & organizational factors by gender on return to work (RTW) in a 5-year follow-up of workers on long-term disability in Quebec (Phase 1)	455 Quebec adults on WC benefits ≥8 weeks at study entry for work-related MSD of the back, neck or upper limbs, 18-60 years old (37% women) (excluded pregnancy, multiple trauma, serious unrelated illness interfering with RTW)	Prospective cohort study with 5-year annual follow-up using WC data linked to telephone interview data	Interview-based time to 1st full or partial RTW lasting ≥3 days, from 2001-2004	Based on WC data, unless "perceived" or "reported" is indicated:<br>- Age<br>- Number of dependents<br>- Annual income<br>- Perceived economic status<br>- Occupational class (blue/white collar)<br>- Perceived physical workload<br>- Hours of paid work/week<br>- Perceived job satisfaction<br>- Reported number of years in curre occupation<br>- Reported job seniority<br>- Reported union membership<br>- Temporary vs. permanent employment status<br>- Company size<br>- Reported 5-item job insecurity ind<br>- Reported awareness of workplace OHS program<br>- Injury site<br>- Type of MSD (back pain, repetitiv strain injury)<br>- WC claim in previous 5 years<br>- Interaction of age & injury site<br>- Interaction of age & type of MSD<br>- Combination of working ≥40 hours/week & having dependents<br>- Combination of working ≥40 hours/week & high perceived physical workload	No additional variables other than prognostic factors	- Survival curves of time to RTW were similar for both genders.<br>- At 2 years, about 40% of both M & W had not attempted a RTW ≥3 days.<br>- At 5 years, this was about 20% for M & W.<br>- In multivariate sex/gender-stratified analyses, poor perceived economic status was associated with longer duration of work absence in both genders<br>- Among women, other risk factors associated with longer duration of work absence were increasing age, combination of working ≥40 hours/week & having dependents, lack of awareness of workplace OHS program & lower income<br>- Among men, other risk factors associated with longer duration of work absence were age >55 years, combination of working ≥40 hours/week & high perceived physical workload & higher job insecurity	Moderate

Study	Study objective(s) (Phase of investigation)	Participants included in MSD-specific analyses	Study design	Work absence outcome(s) measured	Prognostic/ explanatory factor(s) measured	Confounders measured	Results	Study risk of bias
Lederer & Rivard, 2014 [77]	To assess costs, duration & predictors of prolonged compensation benefits by gender in a population characterized by long-term compensation related to musculoskeletal injuries (Phase 1)	22,105 Quebec workers <60 years old on long-term WC wage-replacement benefits ≥3 months for new upper body MSD WC claims accepted between 2001 & 2003 (41% women)	Quebec WC register-based prospective cohort study with 3-year follow-up	Time on WC benefits for an upper body MSD, during 2001-2003 (maximum of 3 years)	- Age at time of injury (computed from birth, 10-year units)<br>- Gross annual personal income pre-injury<br>- Dependents<br>- Size of town of residence<br>- Industry (8 categories)<br>- Type of MSD (traumatic/non-traumatic)<br>- Injury site<br>- Permanent physical impairment<br>- Interaction of age & injury site<br>- Interaction of age & type of MSD<br>- Interaction of type of MSD & presence or not of permanent impairment<br>- Claim history in previous 10 years	No additional variables other than prognostic factors	- 12.3% of M & 7.3% of W absent ≥3 months at recruitment were still on benefits 3 years post-injury. Kaplan-Meier survival curves of proportion on WC benefits over time were similar for M & W, but the rate decreased more slowly among M between years 2 & 3 post-injury.<br>- In SGSA, factors that increased time on benefits in both genders were having an injury with permanent impairment, living in a large city, having dependents, income >$15,000, increasing age & previous claims.<br>- Time-varying effects of age & previous claim history were different between men & women.<br>- Compared to injuries in the upper extremities, injuries in the neck, shoulder/back/trunk & multi-site injuries increased duration on benefits among men, but this was the case only for multi-site injuries in women.	Moderate

Study	Study objective(s) (Phase of investigation)	Participants included in MSD-specific analyses	Study design	Work absence outcome(s) measured	Prognostic/ explanatory factor(s) measured	Confounders measured	Results	Study risk of bias
Smith et al., 2014 [82]	To examine the relationship between age & MSD work injury in British Columbia, Canada. We hypothesize that older age will be associated with more pre-existing chronic conditions & these conditions will mediate the relationship between age & length of sickness absence following an injury, and that the relationship between pre-existing conditions & days of sickness absence will differ across conditions, with the greatest effects for osteoarthritis & mental health conditions (Phase 3)	111,274 British Columbia workers on WC wage-replacement benefits for MSD with injury dates in 1997-1998, 2001-2002, 2005-2006, 15-65 years old (36% women)	Prospective cohort study with 2-year follow-up of WC data linked to provincial health insurance health services data	WC register-based number of days on wage replacement benefits for MSD over 2-year period post-injury	- Age (WC database)<br><br>Hypothesized mediators of age:<br>- 7 pre-existing chronic conditions based on administrative healthcare billing & hospitalization databases, using 5 years of data pre-injury (claimants with 1 hospital or 2 medical records within 2-year period classified as having the condition):<br>o osteoarthritis<br>o rheumatoid arthritis<br>o diabetes<br>o hypertension<br>o coronary heart disease<br>o hearing problems<br>o depression<br>o thyroid conditions	From WC database:<br>- Occupational strength requirements based on job title (light, limited, moderate, heavy)<br>- Occupation involves bending, stooping, kneeling or crouching as primary body position based on job title<br>- Industry (8 categories)<br>- Body part injured<br>- Previous claim in 2 years before current claim<br>- Time period in which injury occurred (1997-1998, 2001-2002, 2005-2006)	- Mean & median number of days on benefits increased with age; the relation was linear among men & linear for women but decreased slightly after age 55. For mean days:<br>o M: 15-24 years: 18 d; 25-34 years: 25 d; 35-44 years: 30 d; 45-54 years: 32 d; ≥55 years: 35 d<br>o W:15-24 years: 25 d; 25-34 years: 37 d; 35-44 years: 42 d; 45-54 years: 42 d; ≥55 years: 38 d<br>- In sex/gender-stratified path analysis models controlling for all confounders, increasing age (continuous) increased the number of days on benefits in both genders. This effect was partly mediated by pre-existing diabetes & depression in both men & women, although the effect of depression was not present among women ≥55 years old.<br>- Indirect effects between age & time on benefits were also present through osteoarthritis in men & coronary heart disease among women.<br>- Pre-existing conditions respectively accounted for 22% & 30% of the effect of age on time on benefits in men & women; 70-78% of the age effect remained unexplained after accounting for pre-existing conditions.	Moderate

M: men, RTW: return to work, W: women

Supplemental Table S5. Description of quantitative studies of explanatory factors of episodes of prolonged work absence for musculoskeletal disorders (MSD) in women and men

Study	Study objective(s) (Phase of investigation)	Participants included in MSD-specific analyses	Study design	Work absence outcome(s) measured	Prognostic/ explanatory factor(s) measured	Confounders measured	Results	Study risk of bias
Kristensen et al., 2007 [74]	To quantify the relations between a range of health & social circumstances experienced throughout childhood & the 4-year risk of medically ascertained musculoskeletal absence from work in young adult age. To study to which degree any factors acting later in life influenced the associations between early factors & later musculoskeletal absence (Phase 3)	378,356 live-born in Norway 1967-1976 eligible for sickness absence benefits in 2000, 27-36 years old (44% women)	Prospective cohort study linking Norwegian National insurance administration (NIA), education register & Norway's Central Population Register data with 27-36 years follow-up starting at birth	1st spell of NIA musculoskeletal sickness absence >16 days 2000-2003	Early-life determinants:<br>- Parental education<br>- Maternal marital status<br>- Mother deceased<br>- Father deceased<br>- Maternal disability pension<br>- Paternal disability pension<br>- Maternal income level (mean above or below absence compensation limit)<br>- Paternal income level (mean above or below absence compensation limit)<br>- Childhood disease (insurance benefit from chronic disease before age 7 years, birth injury or congenital malformation)<br>- Gender-standardized birth weight<br>Hypothesized mediators of early-life determinants:<br>- Subject's educational attainment in 1999<br>- Subject's income in 1999 (gender-specific-quintiles)<br>- Subject's family pattern in 1999 (6 combinations of marital status & children)	- Year of birth (10 categories)<br>- Birth order<br>- Maternal & paternal age at birth<br>- Geographic region of residence at age 16 years	- Cumulative incidence of MSD work absence > 16 days: men: 15.6%, women: 26.4%.<br>- In sex/gender-stratified analyses, parental education level was inversely associated with risk of sickness absence and its effect was partly mediated through own educational attainment in both men & women. Population attributable risk of parental education for MSD sickness absence was 67% for men & 36% for women. Further inclusion of person's income & family pattern had little impact on sickness absence risk & did not affect the population attributable risk.<br>- In women, sickness absence risk was higher for those with childbirth than without & for the former, risk of absence was higher during pregnancy than outside pregnancy (pregnancy-related MSD work absences may explain a some of the sex/gender difference in MSD work absence incidence).	Moderate

Study	Study objective(s) (Phase of investigation)	Participants included in MSD-specific analyses	Study design	Work absence outcome(s) measured	Prognostic/ explanatory factor(s) measured	Confounders measured	Results	Study risk of bias
Laaksonen et al., 2010 [62]	To examine whether: (i) gender differences in sickness absence episodes of various lengths are explained by differences in women's & men's occupations or workplaces *(not specific to MSD)*; (ii) controlling for occupation or workplace has similar effects on gender differences in sickness absence resulting from MSD & other diagnoses; (iii) the contribution of occupation to gender differences in sickness absence is related to vertical differences in women's & men's occupations, i.e. occupational social class *(not specific to MSD)* (Phase 2)	36,395 municipal employees in City of Helsinki, Finland, 18-64 years old (78% women) (excluded single-gender occupations & workplaces, and individuals with absence due to sickness of dependent child or own accident/ injury at work. Maternal or parental leaves subtracted from follow-up time)	Finnish register-based prospective cohort study with 3-year follow-up	Number of medically-confirmed sickness absence episodes >2 weeks for MSDs (from Social Insurance Institution of Finland) 2004-2007	From employer's registers:<br>- Occupation based on job title from City of Helsinki register<br>- Workplace based on City of Helsinki's administrative division of departments & their sub-units<br>- Occupation-workplace combinations	- Age	The age-adjusted female excess in number of episodes of MSD-related sickness absence (rate ratio of 1.87, 95% confidence interval (CI): 1.69-2.08) was attenuated to a similar extent after controlling for either occupation (1.61, 95% CI: 1.36-1.91), workplace (1.61, 1.41-1.84) or occupation-workplace combinations (1.58, 1.32-1.87).	Low

Study	Study objective(s) (Phase of investigation)	Participants included in MSD-specific analyses	Study design	Work absence outcome(s) measured	Prognostic/ explanatory factor(s) measured	Confounders measured	Results	Study risk of bias
Foss et al., 2011 [68]	To determine whether there are gender differences in the effect of exposure to work-related physical and psychosocial risk factors on long-term sickness absence with musculoskeletal diagnoses of more than 8 consecutive weeks<br>(Phase 2)	8,333 Norwegian worker-respondents to the Oslo Health Survey, ages 30, 40 or 45 in 2000-2001, at risk of sickness absence (53% women)	Prospective population-based study with 5-year follow-up linking 2000-2001 Oslo Health Survey data to 2001-2005 Statistics Norway database for sickness absence & education	Having ≥1 register-based episode of long-term sickness absence >8 consecutive weeks with a musculoskeletal diagnosis 2001-2005	From the Oslo Health Survey:<br>- Support from superior (2 items)<br>- Job security (2 items)<br>- Job control (1 item)<br>- Shift work<br>- Physical job demands regarding paid or unpaid work (mainly sedentary/ significant walking/walking & lifting or heavy physical work)<br>- Interaction between psychosocial & physical exposure	- Age (30, 40 or 45 years old)<br>- Education (5 categories, Statistics Norway)	- 5-year cumulative incidence of ≥1 episode of long-term MSD work absence: men: 8.8%, women: 12.6%.<br>- In sex/gender-stratified analyses, compared to sedentary work, heavy physical work or work that involved a lot of walking & lifting was a significant risk factor for ≥1 long-term sickness absence episode in both genders, the effect being strongest among men.<br>- Other significant risk factors in men were low job control & shift/night work or rotating hours, and in women, low support from superior.<br>- Adding the interaction term (psychosocial x physical exposure) to multivariate models did not significantly improve them in either men or women.	Moderate

Study	Study objective(s) (Phase of investigation)	Participants included in MSD-specific analyses	Study design	Work absence outcome(s) measured	Prognostic/ explanatory factor(s) measured	Confounders measured	Results	Study risk of bias
Pekkala et al., 2018 [63]	To examine occupational class differences in long-term sickness absence due to various musculoskeletal diseases among 1.2 million Finnish women and men aged 25–64 years old (Phase 2)	1,280,351 Finnish residents aged 25-64 years old in 2013 (53% women)	Prospective population-based study with one-year follow-up (2014-2015)	1) Having ≥1 registered-based episode of long-term sickness absence >10 days in 2014 for specific ICD-10 MSD categories (M00-M99) measured as receipt of sickness allowance from The Social Insurance Institution of Finland;<br>Back disorders (M40–M54)<br>o Back pain (M54)<br>o Disc disorders (M50–M51)<br>Shoulder disorders (M75)<br>All osteoarthritis (M15–M19)<br>o Knee osteoarthritis (M17)<br>o Hip osteoarthritis (M16)<br>Rheumatoid arthritis (M05–M06)<br>Other musculoskeletal diseases	Occupational class: upper non-manual, lower non-manual, manual	Age	- Proportion of study participants with long-term sickness absence for MSD was higher among women (4.4%) than men (3.1%) for MSDs overall and for the most common diagnoses, e.g. back disorders (W vs. M: 1.7% vs. 1.3%), shoulder disorders (W vs. M: 0.6% vs. 0.5%)<br>- Average length of absence for MSD higher among men (74 days) than women (68 days) for MSDs overall and for the most common diagnoses, e.g. back disorders (M vs. W: 76 vs. 64 days), shoulder disorders (M vs. W: 75 vs. 66 days)<br>- In both genders, age-adjusted relative risk (RR) of new long-term sickness absence episodes was greater among those in lower occupational classes, for MSDs overall (RR was 2-4 times greater) and for all diagnostic categories, the class differences being greatest for back & shoulder disorders: new long-term sickness absence episodes for back pain in manual workers (compared to upper non-manual): W: RR = 4.26 (95% CI:	Low

Study	Study objective(s) (Phase of investigation)	Participants included in MSD-specific analyses	Study design	Work absence outcome(s) measured	Prognostic/ explanatory factor(s) measured	Confounders measured	Results	Study risk of bias
				2) Number of days of sickness absence for MSD among those with an absence episode >10 days			3.90-4.65), M: RR = 5.64 (95% CI: 5.04-6.30). For shoulder disorders, corresponding values are W: RR = 6.06 (95% CI: 5.37-6.84), M: RR = 6.35 (95% CI: 5.51-7.30).<br>- In both genders, age-adjusted length of absence was greater among those in lower occupational classes in all MSD diagnostic categories, the differences being greatest for rheumatoid arthritis & disc disorders in both genders.	

Study	Study objective(s) (Phase of investigation)	Participants included in MSD-specific analyses	Study design	Work absence outcome(s) measured	Prognostic/ explanatory factor(s) measured	Confounders measured	Results	Study risk of bias
Svard et al., 2020 [84]	To examine the association between weight change among normal weight, overweight and obese Finnish public sector employees and subsequent sickness absence due to any musculoskeletal and mental diagnoses (Phase 2)	3,895 municipal employees from Helsinki Health Study cohort who turned 40, 45, 50, 55 or 60 years old between 2000-02 (phase 1), participated in phase 2 in 2007 & consented to data linkage to Social Insurance Institution of Finland database (81% women) (excluded individuals who were pregnant, underweight at phase 1 or retired prior to phase 2 & those with missing info on covariates)	Prospective cohort study with 2007-2013 follow-up (mean: 5.2-year follow-up)	Number of sickness absence spells >9 working days during 2007-2013 follow-up for diagnosed MSD (ICD-10 codes M00–M99) (& other diagnoses not relevant to our review)	Weight change (BMI) ≥5% between phase 1 & 2 (BMI calculated from self-reported height & weight)	- Age<br>- SEP: managers/ professionals, semi-professionals, routine nonmanual, manual<br>- Marital status<br>- Physically strenuous or non-strenuous work<br>- Mentally strenuous or non-strenuous work<br>- Alcohol-related problems (CAGE questionnaire)<br>- Smoking status<br>- Leisure-time physical activity<br>- Prior sickness absence ≥10 days in 1-year period prior to phase 2	- Incidence rate of MSD sickness absence spell >9 working days: women 7.7 vs. men 4.4/100 person-years.<br>- In fully-adjusted sex/gender-stratified analyses, compared to normal weight individuals who maintained their weight, the rate ratio (RR) of MSD sickness absence spells >9 working days was higher in normal weight women who lost weight & in overweight & obese women, regardless of weight gain:<br>o Normal weight/weight loss RR = 1.54, 95% CI: 1.19-1.99<br>o Normal weight/weight gain RR = 1.18, 95% CI: 0.96-1.45<br>o Overweight/stable RR = 1.51, 95% CI: 1.20-1.89<br>o Overweight/gain RR = 1.58, 95% CI: 1.25-2.00<br>o Obese/stable RR = 1.81, 95% CI: 1.33-2.46<br>o Obese/gain RR = 1.75, 95% CI: 1.26-2.44<br>Among men, weight gain in normal weight individuals was associated with reduced rate of MSD sickness absence spells. All other associations weak and ns in fully-adjusted models.	Moderate

Study	Study objective(s) (Phase of investigation)	Participants included in MSD-specific analyses	Study design	Work absence outcome(s) measured	Prognostic/ explanatory factor(s) measured	Confounders measured	Results	Study risk of bias
							○ Normal weight/weight loss RR = 0.96, 95% CI: 0.51-1.82<br>○ Normal weight/weight gain RR = 0.33, 95% CI: 0.15-0.76<br>○ Overweight/stable RR = 1.05, 95% CI: 0.64-1.72<br>○ Overweight/gain RR = 1.04, 95% CI: 0.58-1.87<br>○ Obese/stable RR = 1.10, 95% CI: 0.52-2.32<br>○ Obese/gain RR = 1.72, 95% CI: 0.75-3.93	

CI: confidence interval, ICD: International Classification of Diseases, M: men, ns: not statistically significant, SEP: socioeconomic position, W: women

Supplemental Table S6. Description of quantitative studies of explanatory factors of failure to return to work (RTW) after work absence or prolonged work disability for a musculoskeletal disorder (MSD) in women and men

Study	Study objective(s) (Phase of investigation)	Participants included in MSD-specific analyses	Study design	RTW outcome(s) measured	Prognostic/ explanatory factor(s) measured	Confounders measured	Results	Study risk of bias
Dionne et al., 2007 [67]	To (1) describe the 2-year course of ''return to work in good health'' (RWGH-a composite index of back pain (BP) outcome) among workers who consult in primary care settings for BP with at least 1 day of work absence & (2) identify the determinants of RWGH (failure to RTW) among these subjects (Phase 1)	1,007 Quebec, Canada workers absent from work ≥1 day at time of consultation because of nonspecific BP recruited from 7 primary care settings in QC city area between 1999-2000, 18-64 years old (42% women) (excluded pregnancy, serious co-morbidity affecting work capacity, neck pain & specific causes of BP)	Prospective cohort study with 2-year follow-up	4 categories of RTW according to interview-based occupational status, functional limitations & total number of work absence days since consultation:<br>i. failed RTW<br>ii. failure after RTW attempt<br>iii. partial success (return to regular work + functional limitations ≤ 30% and/or >5 days work absence)<br>iv. successful return to regular work in good health	- 10 sociodemographic factors (e.g. age, education, income, ethnicity, children <5 years old)<br>- BMI<br>- Hand dominance<br>- 7 health behaviour variables (e.g. alcohol consumption, smoking, physical activity, meditation, sleep)<br>- 20 clinical factors/BP history (e.g. functional limitations, previous back surgeries, average pain past 6 months)<br>- 18 health services utilization variables (e.g. number of visits, satisfaction with care, weight of medical file)<br>- 4-item dramatization of diagnosis<br>- 30 occupational factors (e.g. physical job demands, job strain, social support, effort-reward imbalance)<br>- 11 psychological factors (e.g. stressful life events past 12 months, fear-avoidance beliefs (activity; work), health locus of control, pain coping strategies, symptoms of depression); most self-reported	No additional variables other than prognostic factors	At 2 years:<br>- Failed RTW: M: 8.4%, W: 6.2%<br>- Failure after RTW attempt:<br>- M: 10.8%, W: 13.0%<br>- Partial success: M: 22.4%, W: 26.6%<br>- Success: M: 58.5%, W: 54.2%<br>- In multivariate SGSA, RTW failure (compared to success) was associated with fear avoidance beliefs about work in both genders<br>- In women, failure was also associated with persistent pain, pain radiating to extremities, increasing job seniority & fear avoidance beliefs about activity & inversely associated with being unionized & feeling that the physician listened carefully<br>- In men, other risk factors associated with failure included lower age, smoking, poor self-reported health, thoracic pain, high pain level at baseline, perception that job is below qualifications, likelihood of losing job in next 2 years & being on modified duties at baseline & inversely related to receiving WC & dissatisfaction with health services.	Moderate

Study	Study objective(s) (Phase of investigation)	Participants included in MSD-specific analyses	Study design	RTW outcome(s) measured	Prognostic/ explanatory factor(s) measured	Confounders measured	Results	Study risk of bias
Opsahl et al., 2016 [80]	To assess if overall job satisfaction and expectancies of RTW predicts actual RTW after 12 months, among employees with long lasting LBP and to assess if there were gender differences in the predictors (Phase 2)	569 Norwegian employees on sick leave for LBP for 2-10 months, ≥50% sick listed, enrolled in a rehabilitation trial Feb 2008-Aug 2010 (the Cognitive Interventions and Nutritional Supplements trial), 20-60 years old (50% women) (excluded pregnancy, hemophilia, osteoporosis, current cancer, recent back trauma, serious psychiatric disorders)	Prospective cohort study with 1-year follow-up	RTW (full or partial not specified) 12 months after recruitment in trial	- Job satisfaction (1 item)<br>- Own expectancies of RTW (1 item, 4-point response scale)	- Age<br>- Gender<br>- Education<br>- Smoking<br>- Fear avoidance beliefs about LBP (Fear Avoidance Beliefs Questionnaire)<br>- Subjective health complaints<br>- Oswestry back disability index<br>- Emotional distress<br>- Coworker support<br>- Intervention group	- 1-year RTW: M 60.1%, W 52.4%<br>- In analyses adjusting for s/g, men had 1.57 higher odds (95% confidence interval: 1.03–2.40) of RTW compared to women in non-stratified fully-adjusted model<br>- In fully-adjusted SGSA, high expectancies of RTW predicted RTW at 12 months similarly in M & W; high levels of job satisfaction did not.	Moderate

Study	Study objective(s) (Phase of investigation)	Participants included in MSD-specific analyses	Study design	RTW outcome(s) measured	Prognostic/ explanatory factor(s) measured	Confounders measured	Results	Study risk of bias
Maas et al., 2018 [78]	To examine the association between multiple vs. single jobholding and time to RTW for workers with a work-related MSD, stratified by type of MSD, a serious injury indicator, gender, weekly workdays preceding MSD & wage, (Phase 3)	Workers with an accepted MSD lost time claim between 2010 & 2014 in British Columbia, Canada, yielding a matched cohort of 8,389 multiple job holders, i.e. for whom the claim was associated with multiple employers (among 9,029 workers) & 8,389 single job holders (among 125,639 workers) (50% women)	Prospective cohort study with 1-year follow-up	Time to RTW (in calendar days) during 12 months after the 1st time loss day	- Holding multiple jobs (vs. single job)<br><br>Modifying effects tested for:<br>- Gender<br>- Type of MSD, 9 categories:<br>- upper extremity sprains & strains<br>- lower extremity sprains & strains<br>- back (including head, neck, spine, torso) sprains & strains<br>- upper extremity fractures<br>- lower extremity fractures<br>- torso (including head, neck, spine, back) fractures<br>- joint dislocation<br>- dorsopathies<br>- rheumatism excluding the back<br>- Serious injury indicator (yes/no)<br>- Weekly workdays preceding MSD (5 or less vs. 6 or 7)<br>- Annual wage	- Gender<br>- Age<br>- Industry (7 categories)<br>- Occupation (10 categories)<br>- Firm size<br>- History of prior claims (yes/no)<br>- ≥1 accepted claim in preceding 10 years to the MSD claim<br>- Type of MSD<br>- Serious injury indicator<br>- Weekly workdays preceding MSD<br>- Annual wage	- 79.4% of multiple job holders (MJH) returned to work within one year vs. 86.3% of single job holders (SJH).<br>- In analyses adjusted for gender, age, type of MSD, occupation, industry, previous claims, firm size and weekly workdays preceding MSD, MJH were less likely to RTW compared to SJH for the first 6 months during 1-year follow-up (HR = 0.85, 95% CI: 0.78-0.92).<br>- In analyses stratified by the hypothesized effect modifiers, the effect of MJH on RTW was greater among those with a serious injury, higher wage (compared to those on minimum wage, i.e. ≤$20 000), and was seen only for those who worked 5 days or less but not among those working 6 or 7 days/week.<br>- In fully adjusted SGSA, the effect of multiple jobholding on RTW was greater and lasted longer for men than for women: among men, MJH were less likely to RTW compared to SJH for the first 6 months during 1-year follow-up (HR = 0.71, 95% CI: 0.63-0.80); among women, MJH were less likely to RTW compared to SJH for the first 2	Low

Study	Study objective(s) (Phase of investigation)	Participants included in MSD-specific analyses	Study design	RTW outcome(s) measured	Prognostic/ explanatory factor(s) measured	Confounders measured	Results	Study risk of bias
							months after time loss (HR = 0.79, 95% CI: 0.70-0.88).	

Study	Study objective(s) (Phase of investigation)	Participants included in MSD-specific analyses	Study design	RTW outcome(s) measured	Prognostic/ explanatory factor(s) measured	Confounders measured	Results	Study risk of bias
Jones et al., 2021 [60]	To examine the impact of anxiety & depression disorders on sustained RTW for men & women with musculoskeletal strain or sprain. Effect modification of the primary relationship between pre-existing anxiety & depression on sustained RTW by gender was examined on multiplicative & additive scales (Phase 3)	84,925 British Columbia, Canada workers 19-64 years old with accepted lost-time workers' compensation (WC) claims for upper limb or spine strain or sprain between 2009 & 2013, who were registered in the provincial health services registry (42% women) (excluded claims with anxiety or depression healthcare event not meeting case definitions in year before injury, excluded claims with missing data)	Retrospective cohort study with 2-year follow-up that linked provincial WC data with public healthcare system data	Sustained RTW following lost-time episode for upper limb or spine strain or sprain (ICD-9 codes 840, 841, 842, 846, 847) among WC claimants, i.e. number of days from injury date to date of return to non-modified or full job duties & hours (based on pre-injury job duties & hours), with no further wage loss benefits or modified workdays associated with claim for ≥365 days (a full 730 day window was used to ensure sustained RTW)	- Presence of anxiety or depression disorders or both in year prior to MSD injury, based on diagnoses associated with outpatient physician visits, inpatient hospitalizations, and anxiolytic & antidepressant dispensing events registered in the public healthcare records<br>- New onset anxiety & depression disorders occurring after injury but before sustained RTW in workers with no anxiety or depression related healthcare events in the year prior to injury	- Gender<br>- Age group<br>- Income quintile<br>- Dependents in the home<br>- Injured body part<br>- Incident type (exertion, traumatic, fall/slip/trip, contact object, transportation, bodily motion)<br>- Secondary diagnosis on claim<br>- Somatic co-morbidity index score<br>- Other mental co-morbidity (not anxiety or depression; (0, ≥ 1)<br>- Prior claims (0, ≥1)<br>- Firm size<br>- Occupation (10 categories, ex. sales & services, health, management)	- Proportion of women vs. men reaching sustained RTW (non-modified duties) within 1 year: 85.3% vs. 80.0%<br>- In multivariate SGSA, compared to workers with no anxiety or depression in the year prior to injury, workers with one or both pre-existing conditions had lower probability of sustained RTW, the effect size was greater among men than women for anxiety:<br>Men<br>o Pre-existing anxiety HR = 0.88, 95% CI: 0.84–0.93<br>o Pre-existing depression HR = 0.94, 95% CI: 0.89–1.00<br>o Pre-existing anxiety & depression HR = 0.93, 95% CI: 0.90–0.97<br>Women<br>o Pre-existing anxiety HR = 0.95, 95% CI: 0.92–0.99<br>o Pre-existing depression HR = 0.98, 95% CI: 0.93–1.03<br>o Pre-existing anxiety & depression HR = 0.94, 95% CI: 0.91–0.97<br>- In multivariate SGSA, compared to workers with no anxiety or depression in the year prior to injury, workers with one or both pre-existing	Low

Study	Study objective(s) (Phase of investigation)	Participants included in MSD-specific analyses	Study design	RTW outcome(s) measured	Prognostic/ explanatory factor(s) measured	Confounders measured	Results	Study risk of bias
							conditions took longer to RTW: for men the adjusted median was 15, 8, and 8 days longer for those with anxiety, depression or both; corresponding values for women are 5, 2, and 4 days longer compared to those without these pre-existing conditions.<br>- In multivariate SGSA, new-onset anxiety, depression, or both were associated with lower probability of sustained RTW, in both men & women; the effect estimates were greater than for pre-existing conditions:<br>Men<br>o New onset anxiety HR = 0.82, 95% CI: 0.71–0.94<br>o New onset depression HR = 0.69, 95% CI: 0.56–0.85<br>o New onset anxiety & depression HR = 0.63, 95% CI: 0.54–0.74<br>Women<br>o New onset anxiety HR = 0.88, 95% CI: 0.78–1.00<br>o New onset depression HR = 0.72, 95% CI: 0.58–0.90<br>o New onset anxiety & depression HR = 0.65, 95% CI: 0.56–0.74	

Study	Study objective(s) (Phase of investigation)	Participants included in MSD-specific analyses	Study design	RTW outcome(s) measured	Prognostic/ explanatory factor(s) measured	Confounders measured	Results	Study risk of bias
Jones et al., 2023 [61] (sample overlaps Jones et al., 2021)	To examine the impacts of pre-existing anxiety & depression disorders prevalent in the year before injury on the probability of return to non-modified work (from lost-time) & lost-time recurrence after initial return to non-modified work for lost-time upper limb or spine strain or sprain work injury. To examine the differential impacts of these pre-existing disorders on the two phase-based RTW	78,186 British Columbia, Canada workers 19-64 years old with accepted lost-time workers' compensation (WC) claims for upper limb or spine strain or sprain between 2009 & 2013, who were registered in the provincial health services registry for ≥275 days (9 months) in both the year before & the year after injury (41% women)	Retrospective population-based cohort study with 2-year follow-up that linked provincial WC data with public healthcare system data	1) Return to non-modified or full job duties & hours (based on pre-injury duties & hours, with no further wage loss benefits or modified work days associated with claim for ≥365 days) following 1st or 2nd lost-time episode for upper limb or spine strain or sprain (ICD-9 codes 840, 841, 842, 846, 847) among WC claimants, following ≥1 day of lost time (≥7 consecutive calendar days in sensitivity analyses)<br>2) Lost-time recurrence of ≥1 lost workday after initial non-	- Presence of anxiety or depression disorders or both in year prior to MSD injury, based on diagnoses associated with outpatient physician visits, inpatient hospitalizations, and anxiolytic & antidepressant dispensing events registered in the public healthcare records	- Gender<br>- Age group<br>- Income quartile<br>- Dependents in the home<br>- Injured body part<br>- Incident type (exertion, traumatic, fall/slip/trip, contact object, transportation, bodily motion)<br>- Secondary diagnosis on claim<br>- Somatic co-morbidity index score<br>- Other mental co-morbidity (not anxiety or depression)<br>- Prior claims<br>- Firm size<br>- Occupation (10 categories, ex. sales & services, health, management)	- Women more likely than men to RTW (1st RTW 88.5% vs. 84.9%; 2nd RTW 87.8% vs. 81.6%), but also more likely to have a lost-time recurrence after initial RTW (8.5% vs. 6.8%).<br>- In multivariate SGSA, likelihood of RTW pooled across 1st & 2nd RTW events is lower with pre-existing mental health conditions:<br>o 5-10% lower among men with pre-existing anxiety (HR = 0.90, 95% CI: 0.85-0.94) or comorbid anxiety & depression (HR = 0.95, 95% CI: 0.92-0.99) (ns for those with depression only, HR = 0.95, 95% CI: 0.90-1.01)<br>o 4% lower among women with pre-existing anxiety & depression (HR = 0.96, 95% CI: 0.93-0.99) (ns for those with anxiety only (HR = 0.98, 95% CI: 0.94-1.02) or depression only (HR = 1.00, 95% CI:0.95-1.05)).	Low

Study	Study objective(s) (Phase of investigation)	Participants included in MSD-specific analyses	Study design	RTW outcome(s) measured	Prognostic/ explanatory factor(s) measured	Confounders measured	Results	Study risk of bias
	outcomes for both men & women separately (Phase 2)			modified duties RTW (vs. no recurrence)			- In multivariate SGSA, likelihood of lost-time recurrence after initial RTW is higher among those with pre-existing mental health conditions:<br>o 29% higher among men with pre-existing anxiety & depression compared to those without (HR = 1.29, 95% CI: 1.13-1.48) (results ns with anxiety only (HR = 1.12, 95% CI: 0.98-1.42) or depression only (HR = 1.01, 95% CI: 0.81-1.27))<br><br>- 25% higher among women with pre-existing anxiety (HR = 1.25, 95% CI: 1.09-1.43) & 15% higher for those with pre-existing anxiety & depression (HR = 1.15, 95% CI: 1.04-1.28) (result ns with depression alone HR = 1.02, 95% CI: 0.86-1.22)).	

BMI: body mass index, CI: confidence interval, FTE: full-time equivalent employees, HR: hazard ratio, ICD: International Classification of Diseases, (L)BP: (low)back pain, M: men, W: women, ns: not statistically significant, OHS: occupational health & safety, s/g: sex/gender, SGSA: sex/gender-stratified analyses, WC: workers' compensation

Supplemental Table S7. Description of quantitative studies of explanatory factors of receiving social insurance disability pension (DP) for a musculoskeletal disorder (MSD) in women and men

Study	Study objective(s) (Phase of investigation)	Participants included in MSD-specific analyses	Study design	DP outcome(s) measured	Prognostic/ explanatory factor(s) measured	Confounders measured	Results	Risk of bias
Hagen et al., 2000 [71]	To evaluate the influence of education & socioeconomic status on the incidence of disability retirement from back pain (BP)/neck pain (NP) in the general population of Norway (Phase 2)	All employed in Norway, 20-53 years old in 1980 (n=1,333,556) (% women not specified)	Prospective cohort study with 11-year follow-up of NIA DP data & linked census data	Granting of full or partial permanent DP between 1983-1993 for non-inflammatory BP or NP	- Education<br>- Occupational class from 1980 census data (6 categories)	Age	- 11-year cumulative incidence of DP for non-inflammatory BP/NP among all employees was higher among women than men (1.92% vs. 1.42%).<br>- In sex/gender-stratified analyses, lower occupational class, lower education & higher age were associated with higher incidence of DP for non-inflammatory BP/NP in both genders. The effect of education was not mediated by occupational class in stepwise analyses.	Moderate
Hagen et al., 2006 [72]	To examine to what extent the inverse relationship between formal education and disability retirement from BP/NP is mediated by factors related to working conditions and to individual lifestyle (Phase 3)	26,823 survey respondents employed in Nord-Trøndelag county, Norway, 25-59 years old in 1984–1986 (41% women)	Prospective cohort study with 7-year follow-up linking 1984-1986 Nord-Trøndelag Health Survey (HUNT) data with 1987-1993 NIA DP data	Granting of full or partial permanent DP between 1987-1993 for any BP/NP	- Education in 1984 Hypothesized mediators of education (HUNT survey):<br>- Occupational class (3 categories)<br>- Physically demanding work<br>- Concentration at work<br>- Work stress & tension<br>- Authority to plan own work<br>- Job satisfaction<br>- Current smoker<br>- Physical exercise<br>- Measured BMI<br>- Alcohol consumption	Age	- 7-year cumulative incidence of DP for any BP/NP among all employees was higher among women (2.1% vs. 1.7%) than men.<br>- In age-adjusted sex/gender-stratified analyses, each year of formal education was associated with 23-24% decreased risk of DP, equally for men & women. Occupational class & working conditions are mediating factors that contributed the most to educational inequalities in DP for BP/NP in both genders.<br>- For men, in models adjusting for 3 blocks of variables, working conditions reduced the age-adjusted effect of education by 24%, occupational class by 17%, lifestyle by 9%; in women, occupational class, working conditions & lifestyle reduced effect of education equally (by 13% each). In fully-adjusted models, occupational class, working conditions & lifestyle factors reduced the age-adjusted effect of education by 39% for men	Moderate

Study	Study objective(s) (Phase of investigation)	Participants included in MSD-specific analyses	Study design	DP outcome(s) measured	Prognostic/ explanatory factor(s) measured	Confounders measured	Results	Risk of bias
							& by 21% for women. Work stress was not significantly associated with DP in block analyses & was not included in fully-adjusted models.	
Gjesdal et al., 2004 [69]	To retest the importance of several socio-demographic factors among long-term sickness absentees as predictors for DP, using recent data. To investigate whether medical factors also predict transition to DP. To investigate the predictive value of different MSD subgroups (Phase 1)	1,978 sickness absentees >8 weeks with MSD in Hordaland County, Norway, 16-62 years old in 1994 (56% women)	Prospective cohort study with 5-year follow-up of NIS cases of sickness absence >8 weeks during 2 periods in 1994	Granting of full or partial permanent DP for diagnosed MSD (& for other diagnoses not reported in this review) from 1994-1999	- Age<br>- Income in 1993<br>- Unemployment in 1993<br>- Type of doctor<br>- MSD diagnostic subgroup<br>- Treating physician's prediction of prognosis<br>- Number of NIS-compensated sick leave days in 1990-1993	No additional variables other than prognostic factors	- 5-year cumulative incidence of DP for MSD among employees with >8-week work absences was higher among women than men (27.0% vs. 18.0%).<br>- In sex/gender-stratified analyses, higher age, >100 days of previous sickness absence, rheumatoid disease & fibromyalgia/unspecified (compared to fractures & injuries) increased disability risk in both genders, whereas higher income was protective (but ns in men). Among women, the risk of DP was also increased for those with neck disorders & osteoarthritis.	Moderate
Gjesdal et al., 2011 [70]	To assess the chronicity/transition into permanent DP after sickness absence with a MSD and to investigate possible gender differences. If a gender difference is present, to investigate possible socioeconomic & medical	64,249 Norwegians with work absence >8 weeks for MSD, 16-62 years old in 1997 (59% women)	Prospective cohort study with 5-year follow-up of NIS cases of sickness absence >8 weeks 1997-2002	Granting of permanent DP (full or partial not specified) for diagnosed MSD from 1997-2002	- Age<br>- Education<br>- Annual income<br>- Weekly working hours<br>- Not living with children<br>- MSD diagnostic subgroup<br>- Female gender	No additional variables other than prognostic factors	- 5-year cumulative incidence of DP for MSD among employees with >8-week work absences was higher among women than men (22.2% vs. 17.5%). Mean annual incidence per 100 active workers was 4.1 for women & 2.5 for men.<br>- In analyses adjusting for s/g, female excess in disability risk was attenuated & no longer significant when adjusting for age, income, education, part-time work, living with children, rheumatoid arthritis, osteoarthritis, fibromyalgia & neck problems.	Moderate

Study	Study objective(s) (Phase of investigation)	Participants included in MSD-specific analyses	Study design	DP outcome(s) measured	Prognostic/ explanatory factor(s) measured	Confounders measured	Results	Risk of bias
	(biological) explanations (Phase 1)						- In sex/gender-stratified analyses, part-time work, not living with children, fewer years of education, increasing age & lower income increased DP risk in both genders. Rheumatoid arthritis, osteoarthritis & myalgia/fibromyalgia were associated with higher DP risk (compared to back disorders) in both genders, the latter more strongly among women; extremity disorders & fractures/injuries were associated with lower DP risk. Neck disorders were associated with increased risk among men only.	
Vahtera et al., 2010 [85]	To examine whether high worktime control is associated with a reduced risk of subsequent DP independently of other risk factors for ill health (Phase 2)	30,700 Finnish public sector employees, 17-64 years old in 2000-2001 (73% women)	Prospective cohort study linking 2000-2001 survey data from the 10 Towns Finnish Public Sector Study with 2000-2005 Finnish Centre for Pensions data	Granting of permanent or fixed-term, full or partial DP for MSD (& for other diagnoses not reported in this review) between survey year & 2005	- 7-item Self-assessed worktime control<br>- 7-item Co-worker-assessed worktime control (mean work unit scores excluding self-assessment)	- Age<br>- Occupational class (3 categories)<br>- Shift work<br>- Job strain<br>- Effort-reward imbalance<br>- Smoking<br>- Alcohol consumption<br>- Obesity<br>- Leisure-time physical inactivity<br>- Suboptimal self-rated health<br>- Psychological distress	- Mean follow up: 4.4. years<br>- Incidence of DP for MSD was higher among women than men (9.2 vs. 8.7/1000 person-years)<br>- In sex/gender-stratified analyses adjusted for occupational class & age, one unit increase in self-assessed & co-worker-assessed worktime control was associated with decreased risk of DP by 41% & 48%, among men versus 33% & 35% among women. These associations were robust to adjustment for all 17 baseline covariates but s/g-stratified results for full model hazard ratios are not presented.	Moderate

Study	Study objective(s) (Phase of investigation)	Participants included in MSD-specific analyses	Study design	DP outcome(s) measured	Prognostic/ explanatory factor(s) measured	Confounders measured	Results	Risk of bias
						- Prescribed antidepressants<br>- Somatic illness<br>- Mental disorder<br>- Prescribed analgesics<br>- Previous sickness absence		
Mantyniemi et al., 2012 [79]	To investigate the relationship between job strain and all-cause & diagnosis-specific DP in a large cohort of public sector employees (Phase 2)	69,842 public sector employees in the municipal services of 10 Finnish towns & 21 public hospitals (Finnish Public Sector Study) (76% women)	Prospective cohort study linking 2000-2002 survey data to 1994-2005 national health & pension register data (Finnish Centre for Pensions)	Granting of permanent or fixed-term (full time or partial) DP for diagnosed MSD, (ICD-10 codes M00-M99) (& for other diagnoses not reported in this review) between survey year & 2005, mean follow-up 4.6 years	Job strain<br>- Measured by survey with 3 job demand items and 9 job control items from the Job Content Questionnaire<br>- Work unit-based (ex. school or hospital ward)<br>- Occupation-based (within same workplace)<br>- Self-assessed (sensitivity analyses)	Analyses for cause-specific DP only adjusted for:<br>- Age<br>- Type of job contract (temporary/ permanent)	- Incidence of DP for MSD not provided in main analyses; proportion provided in sensitivity analyses: men 1.3% vs. women 1.5%<br>- In sex/gender-stratified analyses, a 1-unit increase in job strain increased risk of DP for MSD to a comparable extent in men & women, regardless of the job strain measure used:<br>- Occupation-based job strain<br>Men HR = 2.41, 95% CI: 1.81-3.21<br>Women HR = 2.21, 95% CI: 1.91-2.57<br>- Work-unit-based job strain<br>Men HR = 1.66, 95% CI: 1.26-2.20<br>Women HR = 1.48, 95% CI: 1.31-1.67<br>- Self-assessed job strain<br>Men HR = 1.44, 95% CI: 1.22-1.70<br>Women HR = 1.33, 95% CI: 1.24-1.43	Moderate

Study	Study objective(s) (Phase of investigation)	Participants included in MSD-specific analyses	Study design	DP outcome(s) measured	Prognostic/ explanatory factor(s) measured	Confounders measured	Results	Risk of bias
Lalluka et al., 2015 [75]	To examine the associations between economic difficulties & subsequent disability retirement among municipal employees from Helsinki. To examine the contribution of other socioeconomic circumstances, health behaviours, working conditions & work-family conflict to the associations between economic difficulties & disability retirement. To focus on disability retirement due to musculoskeletal diseases & mental disorders (Phase 2)	6,170 municipal employees aged 40, 45, 50, 55 or 60 years in 2000-2002 in the City of Helsinki, Finland (78% women)	Prospective cohort study linking 2000-2002 Helsinki Health Survey data to 2000-2010 Finnish Centre for Pensions data	Granting of permanent DP for diagnosed MSD (& for other diagnoses not reported in this review) between survey year & 2010	Current economic difficulties	- Age<br>- Occupational class (5 categories)<br>- Household income divided by household size<br>- Housing tenure (owners vs. renters)<br>- Physical workload<br>- Mental workload<br>- Work–family conflict<br>- Smoking<br>- Alcohol consumption<br>- Obesity	- Cumulative incidence of DP for MSD was higher among women than men (4.4% vs. 2.3%).<br>- Too few cases to allow analysis on male sample.<br>- In women, occasional & frequent economic difficulties (compared to no difficulties) increased risk of DP, when adjusting for age. Additional adjustment for occupational class, household income & housing tenure attenuated these associations and rendered them no longer statistically significant. Further adjustment for health behaviours, and then for working conditions & work-family conflict had but minor effects.	Moderate

Study	Study objective(s) (Phase of investigation)	Participants included in MSD-specific analyses	Study design	DP outcome(s) measured	Prognostic/ explanatory factor(s) measured	Confounders measured	Results	Risk of bias
Kjellberg et al., 2016 [73]	To investigate the association between level of long-term physical workload in middle age and DP before 61 years of age (overall & diagnosis-specific, including for MSD), adjusting for early life factors, educational level & psychosocial working conditions (Phase 2)	11,925 Swedish men and women with complete information on all variables, born in 1948 & 1953, followed during 1991-2009 (47% women)	Prospective cohort study of 2 cohorts of Swedish schoolchildren surveyed for IQ & paternal education in 1961/1966, whose data was linked to register & census data on own education, occupation (1985 & 1990) & DP during 1991-2009	Time to 1st full or partial DP for MSD (ICD-9: 710–739, ICD-10 M01–M99) (& other diagnoses not relevant to this review) during 1991-2009	- Physical workload (based on sex-specific job exposure matrices), applied to the occupation stated in the National Population and Housing Censuses in 1985 & 1990	- Job control (job exposure matrix)<br>- IQ at age 13<br>- Highest educational attainment of the father or male caretaker<br>- Education<br>- Birth cohort	- Proportion of cases receiving DP for MSD during 1991-2009 follow-up was higher among women (7.7%, 433/10,709) than men (3.5%, 224/5,597).<br>- In sex/gender-stratified analyses, stable exposure to high (compared to low) physical workload (measured in both 1985 & 1990) was strongly associated with DP due to MSD during follow-up among both men (crude HR = 5.44, 95% CI: 3.35–8.84) and women (crude HR = 3.82, 95% CI: 2.88–5.08). This increased risk was attenuated after adjustments for paternal education, IQ at age 13, achieved education and job control but it remained two-fold higher among those with high compared to low physical workload (men adjusted HR = 2.25, 95% CI: 1.28–3.94; women adjusted HR = 2.19, 95% CI: 1.48–3.23.	Moderate
Svard et al., 2018 [83]	To examine the joint association of being simultaneously overweight and having a common mental disorder with the risk of subsequent diagnosis-specific disability retirement	6,107 municipal employees who turned 40, 45, 50, 55 or 60 years in 2000-2002 in the City of Helsinki, Finland (78% women)	Prospective cohort study linking 2000-2002 Helsinki Health Survey data to Finnish Centre for Pensions data with	Granting of permanent, temporary or part-time DP for MSD (& for other diagnoses not reported in this review) between survey year and end of follow-up 10 years later	- Being overweight (BMI ≥ 25 kg/m^2)<br>- Having a common mental disorder (GHQ-12≥3).	- Age<br>- SEP: managers/ professionals, semi-professionals, routine nonmanual employees, manual workers<br>- Marital status	- DP for MSD was more common among women (4.3%) than men (2.5%) during mean follow-up of 8.3 years.<br>- In sex/gender-stratified multivariate analyses, risk of DP for MSD was approximately 2-fold higher among overweight women without a common mental disorder and 3-fold higher among overweight women with a common mental disorder, compared to normal-weight women without a common mental disorder. The interaction between BMI and common	Moderate

Study	Study objective(s) (Phase of investigation)	Participants included in MSD-specific analyses	Study design	DP outcome(s) measured	Prognostic/ explanatory factor(s) measured	Confounders measured	Results	Risk of bias
	among Finnish female and male employees (Phase 2)		10-year follow-up			- Physically strenuous or non-strenuous work<br>- Mentally strenuous or non-strenuous work<br>- Problem drinking<br>- Smoking status<br>- Leisure-time physical activity<br>- Presence of a somatic disease	mental disorder was synergistic and dominated by BMI. Corresponding risks in men were 3-fold and 4-fold greater and the interaction between BMI and common mental disorders was antagonistic, suggesting they have independent effects on the risk of DP.<br>- In sensitivity analyses (data not shown), authors report that 16% of women who were both overweight and had a common mental disorder received a DP for MSD if they reported having physically strenuous work vs. 5% if work was physically non-strenuous.	
Siren et al., 2019a [65]	To assess the longitudinal associations of physical and psychosocial work exposures with disability retirement due to a shoulder disorder. To explore whether the associations would differ according to employment sector or industry (Phase 2)	1,135,654 Finns aged 30-59 in 2004, gainfully employed on January 1, 2005 (49% women)	Prospective cohort study with 10-year follow-up (2005-2014)	Granting of full time temporary or permanent DP due to a shoulder disorder (ICD-10 code M75) as the primary diagnosis, between January 1, 2005 & October 31, 2014.	Based on gender-specific job exposure matrices:<br>- Physical work exposures<br>o Heavy physical work<br>o Heavy lifting<br>o Working with hands above shoulder level<br>o Working in forward bent posture<br>o Work requiring high handgrip forces<br>- Psychosocial work exposures<br>o High job demands<br>o Low job control	- Age<br>- Education<br>- Private/public/ other (including self-employed) employment sector<br>- Industry	- Age-adjusted nine-year incidence rate of DP due to shoulder disorder was higher for men than women, respectively 36 cases (95% CI 32–39) and 28 cases (95% CI 25–31) per 100,000 person-years.<br>- In sex/gender-stratified multivariate analyses adjusted for age & education, risk of DP for shoulder disorder was approximately 2- to 3-fold higher among men & women doing physically heavy work. It was also higher in men & women who worked with hands above shoulder level (1.6-fold & 1.2-fold higher, respectively), compared to workers not exposed to these physical work demands. Additionally in men, risk was 1.3-fold higher among those with high job demands, 1.2- to	Low

Study	Study objective(s) (Phase of investigation)	Participants included in MSD-specific analyses	Study design	DP outcome(s) measured	Prognostic/ explanatory factor(s) measured	Confounders measured	Results	Risk of bias
					○ Monotous (repetitive) work<br>○ Job strain categories (Low strain job, Active job, Passive Job, High strain job)		2.1-fold higher among those with low job control and 2- to 3-fold higher among those with high strain jobs, passive jobs and active jobs compared to workers with low strain jobs. Additionally in women, risk of DP was 1.2-fold higher for those working in a forward bent posture, 2- to 3-fold higher among those doing monotonous work, 1.4-fold higher for high strain jobs and lower for active jobs (high demands, high control) (HR = 0.73, 95% CI: 0.55–0.98).<br>- Results were similar in analyses stratified by age and sex/gender, with some exceptions: risk of DP was 1.2-fold higher with exposure to high job demands only among women 50-59 years old, and 1.2-fold higher with low job control only among women 55-59 years old. Risk of DP with exposure to work requiring high handgrip forces varied by age and gender, being higher among younger men 30-44 years old, lower among older men 50-59 years old, lower among younger women 30-44 years old and higher among older women 50-59 years old.<br>- Authors also present analyses stratified by education and by private/public/other employment sector, as well as in 3 main sectors (manufacturing, transportation & storage, health & social work).<br>- Among men, 46% and 49% of cases of DP due to shoulder disorder were respectively attributed to physical and psychosocial work	

Study	Study objective(s) (Phase of investigation)	Participants included in MSD-specific analyses	Study design	DP outcome(s) measured	Prognostic/ explanatory factor(s) measured	Confounders measured	Results	Risk of bias
							exposures. Among women, the corresponding values were 41% and 35%.	
Siren et al., 2019b [64]	To examine the impact of a disabling non-traumatic shoulder disorder on work participation over 9 years and working life expectancy.<br>To study the determinants for (early) preterm exit from paid employment (Phase 1)	7,644 Finns aged 30-59 in 2004, employed or self-employed on 1 January 2006 who had received full-time sickness absence benefit due to shoulder disorder (56% women)	Prospective cohort study with 9-year follow-up (2006-2014)	Preterm (early) exit (before 63 years of age) from paid employment for various causes between 1st day of sickness absence in 2006 & October 31, 2014, after prolonged sickness absence for shoulder disorder (ICD-10 code M75)	- Age<br>- Duration of sickness absence due to shoulder lesion<br>- Education<br>- Region of Finland<br>- Public/private/other sector<br>- Income<br>- Physical work exposures based on gender-specific job exposure matrices<br>o Heavy lifting<br>o Working with hands above shoulder level<br>o Work requiring high handgrip forces<br>- Psychosocial work exposures based on gender-specific job exposure matrices<br>o High job demand<br>o Low job control<br>- Sustained RTW (return to regular duties ≥28 consecutive days)<br>- Participation in vocational rehabilitation (based	- No additional variables other than prognostic factors	- During the 9-year follow-up, 1,523 persons (19.9% of the study population) had a preterm (early) exit, of whom 1,207 (79.0%) received permanent DP.<br>o The primary or secondary diagnosis of DP was shoulder disorder (36%), a spine-related disease (27%), osteoarthritis (23%), mental disorder (16%), neurological disease (8%) & cardiovascular disease (7%). For 74%, the primary or secondary cause was a MSD.<br>- In sex/gender-stratified analyses, predictors of preterm exit common to both genders included older age, >180 days of sickness absence for shoulder disorder, primary level education, no sustained RTW & participation in vocational rehabilitation (association stronger in women).<br>- Predictors in men also included 106-180 days of sickness absence for shoulder disorder.<br>- Predictors in women also included employment in the private sector & heavy lifting.	Low

Study	Study objective(s) (Phase of investigation)	Participants included in MSD-specific analyses	Study design	DP outcome(s) measured	Prognostic/ explanatory factor(s) measured	Confounders measured	Results	Risk of bias
					on administrative/ register data)			
Siren et al., 2020 [66] (same population& sample as Siren et al., 2019a)	To identify occupations with a high risk of disability retirement due to a shoulder disorder in the Finnish working population and to examine whether physical and psychosocial work-related factors explain (mediate) occupational differences in disability retirement (Phase 3)	1,135,654 Finns aged 30-59 in 2004, gainfully employed on January 1, 2005 (49% women)	Prospective cohort study with 10-year follow-up (2005-2014)	Granting of full time temporary or permanent DP due to a shoulder disorder (ICD-10 code M75) as the primary diagnosis, between January 1, 2005 & October 31, 2014.	- Occupation<br>- Physical work exposures (treated as mediators of the effect of occupation) based on gender-specific job exposure matrices:<br>○ Heavy lifting<br>○ Working with hands above shoulder level<br>○ Work requiring high handgrip forces<br>○ Awkward trunk posture<br>○ Physically heavy work<br>- Psychosocial work exposures (treated as mediators of the effect of occupation) based on gender-specific job exposure matrices:<br>○ High job demands<br>○ Low job control<br>○ Monotonous (repetitive) work	- Age<br>- Education	- Age-adjusted nine-year incidence rate of DP due to shoulder disorder was higher for men than women, respectively 36 cases (95% CI 32–39) and 28 cases (95% CI 25–31) per 100,000 person-years.<br>- As compared to professionals, age-adjusted risk of DP was increased among all or most occupational groups in both genders. Among men, highest risk was seen for construction workers, electricians and plumbers (HR = 32.5, 95% CI: 20.7–51.2), then unskilled transport, construction & manufacturing workers (HR = 23.7, 95% CI: 14.5–38.6). Among women, unskilled transport, construction & manufacturing workers had the highest risk (HR = 30.9, 95% CI: 17.2–55.6) then chemical, wood & metal workers (HR = 30.7, 95% CI: 16.2–58.1).<br>- Adjustment for education attenuated the risk as well as the occupational differences in risk in men & women (explaining approximately 2/3 to 3/4 or more of the risk in most occupations).<br>- Further adjustment for physical work exposures explained much of the excess risk of DP in men and women, with heavy physical work showing the largest contribution to the excess risk in both genders & most occupations, while the	Low

Study	Study objective(s) (Phase of investigation)	Participants included in MSD-specific analyses	Study design	DP outcome(s) measured	Prognostic/ explanatory factor(s) measured	Confounders measured	Results	Risk of bias
							contribution of psychosocial work exposures was modest for most occupations.	
Salonen et al., 2020 [81]	To assess how the association between long-term sickness absence and disability retirement varies between diagnostic groups and occupational classes in men and women (Phase 2)	160,088 employed non-retired Finns aged 25-62 in 2006 with a long-term sickness absence spell for MSD starting in 2005 (defined as sickness allowance paid after first 10 consecutive working days have been paid by employer) (59% women)	Prospective cohort study with 8-year follow-up (2007-2014)	Granting of first full or partial DP for MSD (& for other diagnoses not reported in this review) between 2007 & 2014	- Long-term sickness absence for MSD (ICD-10 codes M00-M99) (& other diagnoses not reported in this review)<br>- Occupational class (upper non-manual, lower non-manual, manual, self-employed) treated as a moderator	- Age<br>- Marital status<br>- Urban/rural municipality of residence	- Proportion of women with long-term sickness absence for MSD who transferred to DP for MSD was higher than proportion of men (13.8% vs. 11.2%).<br>- Risk of DP due to MSD was about five times higher among those with MSD-related long-term sickness absence compared to those without MSD absence (who could nevertheless be absent for other diagnoses) (men HR = 5.30, 95% CI: 5.10–5.50; women HR = 4.96, 95% CI: 4.80–5.13).<br>- In analyses further stratified by occupational class, risk of DP for MSD among those with long-term sickness absence for MSD (compared to no MSD absence) was highest among upper non-manual employees in both genders (men HR = 8.76, 95% CI: 7.00–10.96; women HR = 7.50, 95% CI: 6.42–8.77).	Moderate

BP: back pain, DP: disability pension, BMI: body mass index, CI: confidence interval, GHQ: General Health Questionnaire, ICD-10: International Classification of Diseases, 10th revision, HR: hazard ratio, NP: neck pain, NIA (NIS): Norwegian National Insurance Administration (service), SEP: socioeconomic position

Supplemental Table S8. Quality of the evidence on explanatory factors of the duration of work absence/workers' compensation (WC) for a musculoskeletal disorder (MSD) in women and men

Prognostic factors	Number of studies	Number of participants	Multivariate analyses in women	Multivariate analyses in men	Sex/gender differences in association between prognostic factor & duration on benefits (yes/no)	Phase of investigation	GRADE criteria considered when downgrading or upgrading the initial quality of the evidence	Overall quality
Higher age	3<br>Lederer et al., 2012 [76]; Lederer & Rivard, 2014 [77]; Smith et al., 2014 [82]	133,834	+++	+++	No, but different mediators<br>Increasing age is associated with increased duration on benefits in both genders (in Lederer 2012 this is true for men >55 years old); Smith et al: this effect is partly mediated by pre-existing diabetes & depression in both genders, by osteoarthritis in men & by coronary heart disease in women	1 & 3		M: High<br>W: High
Lower income	2<br>Lederer et al., 2012 [76]; Lederer & Rivard, 2014 [77]	22,560	+/-	0/-	Yes<br>In 2012 study, lower income is associated with increased duration of work absence in women; no association in men. In 2014 study, income < $15,000 is associated with decreased duration on benefits in both genders.	1	Downgrade for inconsistency	M: Low<br>W: Low
Poor perceived economic status	1<br>Lederer et al., 2012 [76]	455	+	+	No<br>Poor perceived economic status is associated with increased duration of work absence in both genders	1	Evidence is based on a single study, but not downgraded further due to imprecision	M: Moderate<br>W: Moderate
Job insecurity (index 0-5)	1<br>Lederer et al., 2012 [76]	455	0	+	Yes<br>-Higher job insecurity is associated with increased duration of work absence in men<br>-No association in women	1	Evidence is based on a single study, but not downgraded further due to imprecision	M: Moderate<br>W: Moderate
Having financial dependents (children or spouse)	1<br>Lederer & Rivard, 2014 [77]	22,105	+	+	No<br>Having financial dependents is associated with increased duration on benefits in both genders	1	Downgrade for indirectness (having financial dependents is not necessarily a proxy for caring for dependents)	M: Low<br>W: Low

Prognostic factors	Number of studies	Number of participants	Multivariate analyses in women	Multivariate analyses in men	Sex/gender differences in association between prognostic factor & duration on benefits (yes/no)	Phase of investigation	GRADE criteria considered when downgrading or upgrading the initial quality of the evidence[1]	Overall quality
Working ≥40 hours/week & having dependents	1 Lederer et al., 2012 [76]	455	+	0	Yes<br>-Working ≥40 hours/week & having dependents is associated with increased duration of work absence in women<br>-No association in men	1	Evidence is based on a single study, but not downgraded further due to imprecision	M: Moderate W: Moderate
Working ≥40 hours/week & high physical workload	1 Lederer et al., 2012 [76]	455	0	+	Yes<br>-Working ≥40 hours/week & high perceived physical workload is associated with increased duration of work absence in men<br>-No association in women	1	Evidence is based on a single study, but not downgraded further due to imprecision	M: Moderate W: Moderate
Low job satisfaction	1 Lederer et al., 2012 [76]	455	0	0	No<br>No association in either gender	1	Evidence is based on a single study, but not downgraded further due to imprecision	M: Moderate W: Moderate
Lack of awareness of workplace-based occupational health & safety program	1 Lederer et al., 2012 [76]	455	+	0	Yes<br>-Lack of awareness of workplace-based OHS program is associated with increased duration of work absence in women<br>-No association in men	1	Evidence is based on a single study, but not downgraded further due to imprecision	M: Moderate W: Moderate
Living in a large city	1 Lederer & Rivard, 2014 [77]	22,105	+	+	No<br>Living in a large city (compared to rural or small city) is associated with increased duration on benefits in both genders	1	Evidence is based on a single study, but not downgraded further due to imprecision	M: Moderate W: Moderate
Industry	1 Lederer & Rivard, 2014 [77]	22,105	+	+	Yes<br>-Compared to working in education/public administration, working in other sectors is associated with increased duration on benefits in men<br>-In women, duration is increased in all but the healthcare & transport sectors	1	Evidence is based on a single study, but not downgraded further due to imprecision	M: Moderate W: Moderate

Prognostic factors	Number of studies	Number of participants	Multivariate analyses in women	Multivariate analyses in men	Sex/gender differences in association between prognostic factor & duration on benefits (yes/no)	Phase of investigation	GRADE criteria considered when downgrading or upgrading the initial quality of the evidence[1]	Overall quality
Previous WC claims in the 10 years pre-injury	1<br>Lederer & Rivard, 2014 [77]	22,105	+	+	No<br>A history of previous claims is associated with increased duration on benefits in both genders, but the effect is only significant in the 3rd year post-injury in women	1	Evidence is based on a single study, but not downgraded further due to imprecision	M: Moderate<br>W: Moderate
Interaction between traumatic or non-traumatic MSD & permanent physical impairment	1<br>Lederer & Rivard, 2014 [77]	22,105	+	+	No<br>Having an injury (whether traumatic or not) with permanent physical impairment is associated with increased duration on benefits in both genders	1	Evidence is based on a single study, but not downgraded further due to imprecision	M: Moderate<br>W: Moderate

The quality of the evidence is initially rated high if it is based on phase 2 & 3 studies, and moderate if it is based on phase 1 studies. The evidence can be downgraded due to study limitations, inconsistency, indirectness and publication bias; in the absence of these, it can be upgraded if there are moderate/large effect sizes and dose-response effects. For multivariate analyses, the number of + and - signs respectively represents the number of statistically significant effects with a positive and negative value. A zero indicates that there were no statistically significant effects. M: men, OHS: occupational health and safety, W: women

Supplemental Table S9. Quality of the evidence on explanatory factors of incidence or number of episodes of prolonged work absence for a musculoskeletal disorder (MSD) in women and men

Prognostic factors	Number of studies	Number of participants	Multivariate analyses in women	Multivariate analyses in men	Sex/gender differences in association between prognostic factor & incidence or number of episodes of prolonged MSD work absence (yes/no)	Phase of investigation	GRADE criteria considered when downgrading or upgrading the initial quality of the evidence[1]	Overall quality
Heavy physical work/work involving lots of walking & lifting	1 Foss et al., 2011 [68]	8,333	+	+	No<br>Heavy physical work increases the risk of having ≥1 long-term sickness absence episode, compared to sedentary work, in both genders, the effect being strongest among men	2	Downgrade for study limitations (risk of selection bias) and for imprecision (evidence based on a single study)	M: Moderate W: Moderate
Low job control	1 Foss et al., 2011 [68]	8,333	0	+	Yes<br>-Low job control increases the risk of having ≥1 long-term sickness absence episode in men<br>-No association in women	2	Downgrade for study limitations (risk of selection bias) and for imprecision (evidence based on a single study)	M: Moderate W: Moderate
Shift/night work/rotating hours	1 Foss et al., 2011 [68]	8,333	0	+	Yes<br>-Shift work, night work or rotating hours increase the risk of having ≥1 long-term sickness absence episode in men<br>-No association in women	2	Downgrade for study limitations (risk of selection bias) and for imprecision (evidence based on a single study)	M: Moderate W: Moderate
Low support from superior	1 Foss et al., 2011 [68]	8,333	+	0	Yes<br>-Low support from superior increases the risk of having ≥1 long-term sickness absence episode in women<br>-No association in men	2	Downgrade for study limitations (risk of selection bias) and for imprecision (evidence based on a single study)	M: Moderate W: Moderate
Low job security	1 Foss et al., 2011 [68]	8,333	0	0	No<br>No association in either gender	2	Downgrade for study limitations (risk of selection bias) and for imprecision (evidence based on a single study)	M: Moderate W: Moderate
Lower parental educational attainment	1 Kristensen et al., 2007 [74]	378,356	-	-	No<br>Lower parental educational attainment increases the risk of absence in both genders, the gradient being strongest for men. This effect is partly mediated through own	3	Downgrade for study limitations (unmeasured potential confounding) and for imprecision (evidence based on a single study)	M: Moderate W: Moderate

Prognostic factors	Number of studies	Number of participants	Multivariate analyses in women	Multivariate analyses in men	Sex/gender differences in association between prognostic factor & incidence or number of episodes of prolonged MSD work absence (yes/no)	Phase of investigation	GRADE criteria considered when downgrading or upgrading the initial quality of the evidence[1]	Overall quality
					educational attainment, and mediated to a lesser extent by own income or family pattern			
Occupation	1 Laaksonen et al., 2010 [62]	36,395	+	+	Yes<br>There is an age-adjusted female excess in number of episodes of MSD-related sickness absence that was attenuated after controlling for occupation	2	Downgrade for imprecision (evidence is based on a single study)	M: Moderate W: Moderate
Workplace	1 Laaksonen et al., 2010 [62]	36,395	+	+	Yes<br>There is an age-adjusted female excess in number of episodes of MSD-related sickness absence that was attenuated after controlling for workplace	2	Downgrade for imprecision (evidence is based on a single study)	M: Moderate W: Moderate
Occupation-workplace combinations	1 Laaksonen et al., 2010 [62]	36,395	+	+	Yes<br>There is an age-adjusted female excess in number of episodes of MSD-related sickness absence that was attenuated after controlling for occupation-workplace combinations	2	Downgrade for imprecision (evidence is based on a single study)	M: Moderate W: Moderate
Occupational class (upper non-manual, lower non-manual, manual)	1 Pekkala et al., 2018 [63]	1,280,351	+	+	No<br>In both genders, age-adjusted incidence of long-term sickness absence for MSD was 2-4 times greater among those in lower occupational classes (manual & lower non-manual) compared to the highest occupational class, i.e. upper non-manual	2	Downgrade for study limitations (unmeasured potential confounding) and for imprecision (evidence based on a single study)	M: Moderate W: Moderate
Weight change (BMI) ≥5%	1 Svard et al., 2020 [84]	3,895	+	-	Yes<br>-Among women, weight loss was associated with higher rate of MSD sickness absence spells >9 working days. Weight gain had no additional effect on the already higher rate among overweight & obese women, compared to normal weight individuals who maintained their weight.	2	Downgrade for study limitations (validity of prognostic factor, which is based on self-reported BMI) and for imprecision (evidence based on a single study)	M: Moderate W: Moderate

Prognostic factors	Number of studies	Number of participants	Multivariate analyses in women	Multivariate analyses in men	Sex/gender differences in association between prognostic factor & incidence or number of episodes of prolonged MSD work absence (yes/no)	Phase of investigation	GRADE criteria considered when downgrading or upgrading the initial quality of the evidence[1]	Overall quality
					-Among men, weight gain in normal weight individuals was associated with reduced rate of MSD sickness absence spells.			

[1]The quality of the evidence is initially rated high if it is based on phase 2 & 3 studies, and moderate if it is based on phase 1 studies. The evidence can be downgraded due to study limitations, inconsistency, indirectness and publication bias; in the absence of these, it can be upgraded if there are moderate/large effect sizes and dose-response effects. For multivariate analyses, the number of + and - signs respectively represents the number of statistically significant effects with a positive and negative value. A zero indicates that there were no statistically significant effects. M: men, W: women

Supplemental Table S10. Quality of the evidence on explanatory factors of failure to return to work (RTW) following work absence for a musculoskeletal disorder (MSD) in women and men

Prognostic factors	Number of studies	Number of participants	Multivariate analyses in women	Multivariate analyses in men	Sex/gender differences in association between prognostic factor & failure to RTW (yes/no)	Phase of investigation	GRADE criteria considered when downgrading or upgrading the initial quality of the evidence[1]	Overall quality
Lower age	1 Dionne et al., 2007 [67]	1,007	0	+	Yes<br>-Decreasing age is associated with failure to RTW in men<br>-Age is not associated with RTW in women	1	Evidence is based on a single study, but not downgraded further due to imprecision	M: Moderate W: Moderate
Current financial problems because of BP	1 Dionne et al., 2007 [67]	1,007	0	0	No<br>No association in either gender	1	Evidence is based on a single study, but not downgraded further due to imprecision	M: Moderate W: Moderate
Smoking	1 Dionne et al., 2007 [67]	1,007	0	+	Yes<br>-Smoking is associated with failure to RTW in men<br>-No association in women	1	Evidence is based on a single study, but not downgraded further due to imprecision	M: Moderate W: Moderate
High baseline pain level (5-10 vs. 0-4)	1 Dionne et al., 2007 [67]	1,007	0	+	Yes<br>-Higher baseline pain level is associated with failure to RTW in men<br>- No association in women	1	Evidence is based on a single study, but not downgraded further due to imprecision	M: Moderate W: Moderate
Persistent BP episode	1 Dionne et al., 2007 [67]	1,007	+	0	Yes<br>-Persistent BP episode is associated with failure to RTW in women<br>- No association in men	1	Evidence is based on a single study, but not downgraded further due to imprecision	M: Moderate W: Moderate
≥1 previous BP surgeries	1 Dionne et al., 2007 [67]	1,007	0	+	Yes<br>-≥1 previous BP surgery is associated with failure to RTW in men<br>- No association in women	1	Evidence is based on a single study, but not downgraded further due to imprecision	M: Moderate W: Moderate
Pain radiating to upper or lower limb	1 Dionne et al., 2007 [67]	1,007	+	0	Yes<br>-Radiating pain is associated with failure to RTW in women<br>- No association in men	1	Evidence is based on a single study, but not downgraded further due to imprecision	M: Moderate W: Moderate
Poor self-reported health status	1 Dionne et al., 2007 [67]	1,007	0	+	Yes<br>-Poor self-reported health is associated with failure to RTW in men<br>- No association in women	1	Evidence is based on a single study, but not downgraded further due to imprecision	M: Moderate W: Moderate

Prognostic factors	Number of studies	Number of participants	Multivariate analyses in women	Multivariate analyses in men	Sex/gender differences in association between prognostic factor & failure to RTW (yes/no)	Phase of investigation	GRADE criteria considered when downgrading or upgrading the initial quality of the evidence[1]	Overall quality
Thoracic or cervico-thoracic pain site	1 Dionne et al., 2007 [67]	1,007	0	+	Yes<br>-Thoracic or cervico-thoracic pain compared to thoraco-lumbar/lumbar/sacral pain is associated with failure to RTW in men<br>-No association in women	1	Evidence is based on a single study, but not downgraded further due to imprecision	M: Moderate W: Moderate
Current functional limitations	1 Dionne et al., 2007 [67]	1,007	0	0	No<br>No association in either gender	1	Evidence is based on a single study, but not downgraded further due to imprecision	M: Moderate W: Moderate
Currently receiving workers' compensation (WC) for BP	1 Dionne et al., 2007 [67]	1,007	0	-	Yes<br>-Receiving WC for BP is inversely associated with failure to RTW in men<br>-No association in women	1	Evidence is based on a single study, but not downgraded further due to imprecision	M: Moderate W: Moderate
Dissatisfaction with health services since consultation	1 Dionne et al., 2007 [67]	1,007	0	-	Yes<br>-Dissatisfaction with healthcare services is inversely associated with failure to RTW in men<br>-No association in women	1	Evidence is based on a single study, but not downgraded further due to imprecision	M: Moderate W: Moderate
"The doctor did not listen carefully while you described your back problem"	1 Dionne et al., 2007 [67]	1,007	+	0	Yes<br>-Feeling that the doctor did not listen carefully is inversely associated with failure to RTW in women<br>-No association in men	1	Evidence is based on a single study, but not downgraded further due to imprecision	M: Moderate W: Moderate
Perception that doctor's diagnosis of back problem is correct	1 Dionne et al., 2007 [67]	1,007	0	0	No<br>No association in either gender	1	Evidence is based on a single study, but not downgraded further due to imprecision	M: Moderate W: Moderate
"Your doctor, physical therapist or another professional showed you how to work to protect	1 Dionne et al., 2007 [67]	1,007	0	0	No<br>No association in either gender	1	Evidence is based on a single study, but not downgraded further due to imprecision	M: Moderate W: Moderate

Prognostic factors	Number of studies	Number of participants	Multivariate analyses in women	Multivariate analyses in men	Sex/gender differences in association between prognostic factor & failure to RTW (yes/no)	Phase of investigation	GRADE criteria considered when downgrading or upgrading the initial quality of the evidence[1]	Overall quality
your back from injury”								
Workload index (unit) (frequency x intensity of effort)	1 Dionne et al., 2007 [67]	1,007	0	0	No<br>No association in either gender	1	Evidence is based on a single study, but not downgraded further due to imprecision	M: Moderate W: Moderate
High work pace	1 Dionne et al., 2007 [67]	1,007	0	0	No<br>No association in either gender	1	Evidence is based on a single study, but not downgraded further due to imprecision	M: Moderate W: Moderate
Perception that occupation is below qualifications	1 Dionne et al., 2007 [67]	1,007	0	+	Yes<br>-The perception that job is below qualifications is associated with failure to RTW in men<br>-No association in women	1	Evidence is based on a single study, but not downgraded further due to imprecision	M: Moderate W: Moderate
Higher job seniority	1 Dionne et al., 2007 [67]	1,007	+	0	Yes<br>-Increasing job seniority is associated with failure to RTW in women<br>-No association in men	1	Evidence is based on a single study, but not downgraded further due to imprecision	M: Moderate W: Moderate
Low job satisfaction	1 Opsahl et al., 2016 [80]	569	0	0	No<br>No association in either gender	2	Downgrade for study limitations (risk of selection bias) and for imprecision (evidence based on a single study)	M: Moderate W: Moderate
Likelihood of losing job next 2 years	1 Dionne et al., 2007 [67]	1,007	0	+	Yes<br>-Likelihood of losing job in the next 2 years is associated with failure to RTW in men<br>-No association in women	1	Evidence is based on a single study, but not downgraded further due to imprecision	M: Moderate W: Moderate
Unionized job	1 Dionne et al., 2007 [67]	1,007	-	0	Yes<br>-Being unionized is inversely associated with failure to RTW in women<br>-No association in men	1	Evidence is based on a single study, but not downgraded further due to imprecision	M: Moderate W: Moderate

Prognostic factors	Number of studies	Number of participants	Multivariate analyses in women	Multivariate analyses in men	Sex/gender differences in association between prognostic factor & failure to RTW (yes/no)	Phase of investigation	GRADE criteria considered when downgrading or upgrading the initial quality of the evidence[1]	Overall quality
Being on modified duties at consultation	1 Dionne et al., 2007 [67]	1,007	0	+	Yes<br>-Being on modified duties at baseline is associated with failure to RTW in men<br>-No association in women	1	Evidence is based on a single study, but not downgraded further due to imprecision	M: Moderate W: Moderate
Fear avoidance beliefs about activity	1 Dionne et al., 2007 [67]	1,007	+	0	Yes<br>-Fear avoidance beliefs about activity are associated with failure to RTW in women<br>-No association in men	1	Evidence is based on a single study, but not downgraded further due to imprecision	M: Moderate W: Moderate
Fear avoidance beliefs about work	1 Dionne et al., 2007 [67]	1,007	+	+	No<br>Fear avoidance beliefs about work are associated with failure to RTW in both genders	1	Evidence is based on a single study, but not downgraded further due to imprecision	M: Moderate W: Moderate
Low expectancies of RTW	1 Opsahl et al., 2016 [80]	569	+	+	No<br>Low expectancy of RTW is associated with failure to RTW in both genders	2	Downgrade for study limitations (risk of selection bias) and for imprecision (evidence based on a single study)	M: Moderate W: Moderate
Symptoms of depression in the past month	1 Dionne et al., 2007 [67]	1,007	0	0	No<br>No association in either gender	1	Evidence is based on a single study, but not downgraded further due to imprecision	M: Moderate W: Moderate
Holding multiple jobs (vs. single job)	1 Maas et al., 2018 [78]	16,778	+	+	No<br>Multiple job holding is associated with reduced likelihood to RTW after time loss MSD in both genders, but the result holds for the first 6 months in men and for the first two months in women during 1-year follow-up	3	Downgrade for study limitations (unmeasured potential confounding) & for imprecision (evidence is based on a single study)	M: Moderate W: Moderate
Pre-existing anxiety disorder	1 Jones et al., 2021 [60]; 2023 [61]*	84,925	0	+	Yes & no, depending on specific RTW outcome:<br>Yes: Pre-existing anxiety is associated with lower likelihood to RTW in men, but association weaker or not statistically significant in women, depending on 2021 or 2023 study;	2 & 3	Downgrade for imprecision (evidence is based on a single study)	M: Moderate W: Moderate

Prognostic factors	Number of studies	Number of participants	Multivariate analyses in women	Multivariate analyses in men	Sex/gender differences in association between prognostic factor & failure to RTW (yes/no)	Phase of investigation	GRADE criteria considered when downgrading or upgrading the initial quality of the evidence[1]	Overall quality
					No: pre-existing anxiety increased likelihood of lost-time recurrence after initial RTW to a similar extent in both genders			
Pre-existing depression disorder	1 Jones et al., 2021 [60]; 2023 [61]*	84,925	0	0	No Pre-existing depression is not associated with lower likelihood to RTW in either gender	2 & 3	Downgrade for imprecision (evidence is based on a single study)	M: Moderate W: Moderate
Pre-existing anxiety & depression disorders	1 Jones et al., 2021 [60]; 2023 [61]*	84,925	+	+	No Pre-existing anxiety & depression are associated with lower likelihood to RTW in both genders	2 & 3	Downgrade for imprecision (evidence is based on a single study)	M: Moderate W: Moderate
New-onset anxiety disorder	1 Jones et al., 2021 [60]	84,925	+	+	No New-onset anxiety is associated with lower likelihood to RTW in both genders	3	Downgrade for imprecision (evidence is based on a single study)	M: Moderate W: Moderate
New-onset depression disorder	1 Jones et al., 2021 [60]	84,925	+	+	No New-onset depression is associated with lower likelihood to RTW in both genders	3	Downgrade for imprecision (evidence is based on a single study)	M: Moderate W: Moderate
New-onset anxiety & depression disorders	1 Jones et al., 2021 [60]	84,925	+	+	No New-onset anxiety & depression is associated with lower likelihood to RTW in both genders	3	Downgrade for imprecision (evidence is based on a single study)	M: Moderate W: Moderate

[1]The quality of the evidence is initially rated high if it is based on phase 2 & 3 studies, and moderate if it is based on phase 1 studies. The evidence can be downgraded due to study limitations, inconsistency, indirectness and publication bias; in the absence of these, it can be upgraded if there are moderate/large effect sizes and dose-response effects. For multivariate analyses, the number of + and - signs respectively represents the number of significant effects with a positive and negative value. A zero indicates that there were no significant effects. M: men, W: women; *Jones et al., 2021 & 2023 have overlapping samples and are counted as one study for the purposes of grading the quality of the evidence.

Supplemental Table S11. Quality of the evidence on explanatory factors of receiving social insurance disability pension (DP) for a musculoskeletal disorder (MSD) in women and men

Prognostic factors	Number of studies	Number of participants	Multivariate analyses in women	Multivariate analyses in men	Sex/gender differences in association between prognostic factor & DP (yes/no)	Phase of investigation	GRADE criteria considered when downgrading or upgrading the initial quality of the evidence[1]	Overall quality
Higher age	4 Hagen et al., 2000 [71]; Gjesdal et al., 2004 [69]; 2011 [70]; Siren et al., 2019b [64]	1,407,427	++++	++++	No Increasing age increases the risk of DP in both genders	1 & 2	Downgrade for study limitations (unmeasured potential confounding) Upgrade for dose-response & large effect size	M: High W: High
Lower education	4 Hagen et al., 2000 [71]; 2006 [72]; Gjesdal et al., 2011 [70]; Siren et al., 2019b [64]	1,432,272	++++	++++	No Lower education increases the risk of DP in both genders; the effect may be mediated more so by working conditions in men, and in women, by an equal contribution of working conditions, occupational class & lifestyle. Altogether, these factors mediate 39% of the effect in men & 21% in women	1, 2 & 3	Downgrade for study limitations (unmeasured potential confounding)	M: Moderate W: Moderate
Lower occupational class	2 Hagen et al., 2000 [71]; Salonen et al., 2020 [81]	1,493,644	++	++	No Lower occupational class increases the risk of DP in both genders	2	Downgrade for study limitations (unmeasured potential confounding)	M: Moderate W: Moderate
Occupation	1 Siren et al., 2020 [66]	1,135,654	+	+	No Compared to professionals, age-adjusted risk of DP was increased among all or most occupational groups in both genders, and the excess risk of DP after	3	Downgrade for study limitations (unmeasured potential confounding) & for imprecision (evidence is based on a single study)	M: Moderate W: Moderate

					further adjustment for education was mainly mediated by physical work exposures, while the contribution of psychosocial work exposures was modest, in both genders.			
Private (vs. public) employment sector	1 Siren et al., 2019b [64]	7,644	+	0	Yes Working in the private sector increases the risk of preterm (early) exit from paid employment (most of which is DP) in women, but not men.	1	Evidence is based on a single study, but not downgraded further due to imprecision	M: Moderate W: Moderate
Lower income	3 Gjesdal et al., 2004 [69]; 2011 [70]; Siren et al., 2019b [64]	73,871	++/0	++/0	No Lower income increases the risk of DP in both genders in two studies, but has no effect on risk of preterm (early) exit from paid employment (most of which is DP) in either gender in the third study	1	Downgrade for study limitations (unmeasured potential confounding) ~~& inconsistency~~	M: Low W: Low
Unemployment	1 Gjesdal et al., 2004 [69]	1,978	0	0	No No association between unemployment & risk of DP	1	Downgrade for study limitations (unmeasured potential confounders) & imprecision (evidence is based on a single study)	M: Low W: Low
Economic difficulties in paying bills & buying food/clothes	1 Lalluka et al., 2015 [75]	6,170	+	n/a	N/A Only women were analyzed: economic difficulties increase the risk of DP	2	Downgrade for imprecision (evidence is based on a single study)	M: Moderate W: Moderate
Part-time work	1 Gjesdal et al., 2011 [70]	64,249	+	+	No Part-time work increases the risk of DP in both genders	1	Downgrade for study limitations (unmeasured potential confounders) & imprecision (evidence is based on a single study)	M: Low W: Low
Greater duration of sickness absence for MSD	3 Gjesdal et al., 2004 [69]; Siren et al., 2019b [64]; Salonen et	169,710	+++	+++	No Greater duration of sickness absence for MSD increases the risk of DP in both genders	1 & 2	Downgrade for study limitations (unmeasured potential confounders)	M: Moderate W: Moderate

	al., 2020 [81]							
Living with children	1 Gjesdal et al., 2011 [70]	64,249	+	+	No<br>Risk of DP is higher for those who do not live with children in both genders	1	Downgrade for study limitations (unmeasured potential confounders) & imprecision (evidence is based on a single study)	M: Low<br>W: Low
Being overweight (BMI ≥25 kg/m2)	1 Svard et al., 2018 [83]	6,107	+	+	No<br>Being overweight increases the risk of DP in both genders. The risk is approximately 2-fold higher among overweight women without a common mental disorder and 3-fold higher among overweight women with a common mental disorder, compared to normal-weight women without a common mental disorder. The interaction between BMI and common mental disorder was synergistic and dominated by BMI. Corresponding risks in men were 3-fold and 4-fold greater and the interaction between BMI and common mental disorders was antagonistic, suggesting they have independent effects on the risk of DP.	2	Downgrade for study limitations (self-reported BMI) & imprecision (evidence is based on a single study)<br>Upgrade for strong effect sizes	M: Moderate<br>W: Moderate
Common mental disorder at baseline (GHQ-12≥3)	1 Svard et al., 2018 [83]	6,107	+	+	No<br>Having a common mental disorder increases the risk of DP in both genders, but in women, its effect is synergistic to that of being overweight, while in men its effect is antagonistic, suggesting it independently increases risk of DP.	2	Downgrade for study limitations (self-reported BMI) & imprecision (evidence is based on a single study)<br>Upgrade for strong effect sizes	M: Moderate<br>W: Moderate
High job demands	2 Siren et al., 2019a [65], 2019b [64]	1,143,298	+/0	+/0	Yes<br>In one study, no association of high job demands with risk of preterm (early) exit from paid employment. In the other study, risk of DP is higher with exposure to high job demands in men across all age groups & educational levels studied,	1 & 2	Downgrade for study limitations (unmeasured potential confounding & potential exposure misclassification related to use of job exposure matrix)	M: Moderate<br>W: Moderate

					the private sector (& other/self-employed) but not the public sector, and manufacturing but not transportation & storage sectors; in women, risk of DP was higher with exposure to high job demands only among those 50-59 years old, and those with secondary level education; no associations seen in analyses stratified by private/public sector; risk was lower among women in health & social work sector, no association seen in manufacturing sector			
Low job control	2 Siren et al., 2019a [65], 2019b [64]	1,143,298	+/0	+/0	No In one study, no association of low job control with risk of preterm (early) exit from paid employment. In the other study, low job control increases the risk of DP in both genders (in men, association to DP holds for those aged 30-59 years old, in women, association holds only among those 55-59 years old); risk was higher among men in manufacturing but not transportation & storage, and not in women in the industries examined.	1 & 2	Downgrade for study limitations (unmeasured potential confounding & potential exposure misclassification related to use of job exposure matrix)	M: Moderate W:Moderate
Lower self-assessed worktime control	1 Vahtera et al., 2010 [85]	30,700	+	+	No Lower worktime control increases the risk of DP in both genders	2	Downgrade for imprecision (evidence is based on a single study)	M: Moderate W: Moderate
Lower coworker-assessed worktime control	1 Vahtera et al., 2010 [85]	30,700	+	+	No Lower worktime control increases the risk of DP in both genders	2	Downgrade for imprecision (evidence is based on a single study)	M: Moderate W: Moderate
Monotonous (repetitive) work	1 Siren et al., 2019a [65]	1,135,654	+	0	Yes Risk of DP is higher among women doing monotonous work, but not men overall; risk was higher among women in manufacturing and health & social	2	Downgrade for study limitations (unmeasured potential confounding & potential exposure misclassification related to use of job exposure matrix) & for imprecision	M: Moderate W: Moderate

					work, but not in men in the industries examined		(evidence is based on a single study)	
High strain job (high demands, low control)	2 Mantyniemi et al., 2012 [79]; Siren et al., 2019a [65]	1,205,496	++	++	No<br>Risk of DP is higher among those with high strain jobs in both genders to a comparable extent, regardless of the job strain measure used (quadrant or subtraction approach, occupation-based, unit-based or self-assessed job strain)	2	Downgrade for study limitations (unmeasured potential confounding & potential for exposure misclassification)	M: Moderate W: Moderate
Active job (high demands, high control)	1 Siren et al., 2019a [65]	1,135,654	-	+	Yes<br>Risk of DP is higher among men and lower among women in "active" jobs compared to those in low strain jobs (low demands, high control)	2	Downgrade for study limitations (unmeasured potential confounding & potential exposure misclassification related to use of job exposure matrix) & for imprecision (evidence is based on a single study)	M: Moderate W: Moderate
Passive job (low demands, low control)	1 Siren et al., 2019a [65]	1,135,654	0	+	Yes<br>Risk of DP is higher among men in "passive" jobs, compared to men in low strain jobs (low demands, high control); no association in women in fully-adjusted model	2	Downgrade for study limitations (unmeasured potential confounding & potential exposure misclassification related to use of job exposure matrix) & for imprecision (evidence is based on a single study)	M: Moderate W: Moderate
Heavy physical work	2 Siren et al., 2019a [65]; Kjellberg et al., 2016 [73]	1,147,579	++	++	No<br>Risk of DP is higher with physically heavy work in both genders overall; risk is higher in men in manufacturing, but not transportation & storage, and higher in women in manufacturing and health & social work	2	Downgrade for study limitations (unmeasured potential confounding & potential exposure misclassification related to use of job exposure matrix)	M: Moderate W: Moderate
Manual handling of heavy loads (heavy lifting)	2 Siren et al., 2019a [65], 2019b [64]	1,143,298	++	+/0	Yes<br>In one study, heavy lifting increases risk of preterm (early) exit from paid employment in women, but not men. In the other study, heavy lifting increases risk of DP among women 50-54 years old but is associated with lower risk among women in the public sector and those 30-49 years old, no associations in industry-stratified analyses in women; in	1 & 2	Downgrade for study limitations (unmeasured potential confounding & potential exposure misclassification related to use of job exposure matrix)	M: Moderate W: Moderate

					men, no association overall in fully-adjusted models; in stratified models, heavy lifting increases risk of DP among men in the private sector, in transportation & storage, but not in manufacturing, but risk of DP with heavy lifting is lower among men 50-54 years old			
Working with hands above shoulder level	2 Siren et al., 2019a [65], 2019b [64]	1,143,298	+/0	+/0	No<br>In one study, no association with risk of preterm (early) exit from paid employment in either gender. In the other study, working with hands above shoulder level increases risk of DP in both genders (association is largely consistent in analyses stratified by age, education, & for men, by private/public/other sector); in analyses stratified by industry, risk is higher among men in transportation & storage, but not in manufacturing, nor among women in the industries examined	1 & 2	Downgrade for study limitations (unmeasured potential confounding & potential exposure misclassification related to use of job exposure matrix)	M: Moderate W: Moderate
Working in forward bent posture	1 Siren et al., 2019a [65]	1,135,654	+	+	No<br>Risk of DP is higher for those working in a forward bent posture among women 30-49 years old & men 50-59 years old; no associations in industry-stratified analyses	2	Downgrade for study limitations (unmeasured potential confounding & potential exposure misclassification related to use of job exposure matrix) & for imprecision (evidence is based on a single study)	M: Moderate W: Moderate
Work requiring high handgrip forces	2 Siren et al., 2019a [65], 2019b [64]	1,143,298	+/0	+/0	Yes<br>In one study, no association with risk of preterm (early) exit from paid employment in either gender. In the other study, risk of DP with work requiring high handgrip forces varies by age, education and gender: it is higher among younger men 30-44 years old & lower in older men 50-59 years old; among women, risk is lower among	1 & 2	Downgrade for study limitations (unmeasured potential confounding & & potential exposure misclassification related to use of job exposure matrix)	M: Moderate W: Moderate

					those 30-44 years old & higher among those 50-59 years old; in analyses stratified by education, risk is higher in men with tertiary level education & women with primary & secondary education; no associations in analyses stratified by private/public/other employment sector or by industry			
Sustained RTW (return to regular duties ≥28 consecutive days)	1 Siren et al., 2019b [64]	7,644	+	+	No<br>Sustained RTW decreases the risk of preterm (early) exit from paid employment in both genders	1	Evidence is based on a single study, but not downgraded further due to imprecision	M: Moderate W: Moderate
Participation in vocational rehabilitation	1 Siren et al., 2019b [64]	7,644	+	+	No<br>Participation in vocational rehabilitation increases the risk of preterm (early) exit from paid employment in both genders (this association is stronger among women)	1	Evidence is based on a single study, but not downgraded further due to imprecision	M: Moderate W: Moderate

[1]The quality of the evidence is initially rated high if it is based on phase 2 & 3 studies, and moderate if it is based on phase 1 studies. The evidence can be downgraded due to study limitations, inconsistency, indirectness and publication bias; in the absence of these, it can be upgraded if there are moderate/large effect sizes and dose-response effects. For multivariate analyses, the number of + and - signs respectively represents the number of significant effects with a positive and negative value. A zero indicates that there were no significant effects.
BMI: body mass index, GHQ: General Health Questionnaire, M: men, N/A: not applicable, RTW: return to work, W: women

Supplemental Table S12. Description of qualitative studies of factors influencing return to work and/or stay at work of women and men with musculoskeletal disorders (MSD)

Study	Study objective(s)	Participants analyzed	Study design/method	Study quality
Ahlgren & Hammarström, 2000 [93]	To explore experiences in the rehabilitation process in order to find possible explanations for the fact that women are less likely to return to work than are men.	11 Swedish blue-collar workers <30 years old with work-related MSD, married/cohabiting, with children, most of whom had completed vocational rehabilitation at the time of interview in 1995, strategically sampled from 1994 quantitative study on vocational rehabilitation (64% women)	- 1-2-hour interviews with open-ended questions about the rehabilitation process<br>- Men & women's experiences were compared on: i) cause of injury, ii) diagnosis, iii) negotiations for a new work situation & iv) goals hoped to be achieved through the rehabilitation process<br>- Participants validated interview transcripts.<br>- Only the perspective of the workers was explored. No triangulation with other parties.<br>- Most of the doctors and social security officers were men.	Medium
Liedberg & Henriksson, 2002 [94]	To examine which factors women with fibromyalgia (FM) perceive as influencing their capacity to remain in a work role.	39 Swedish women from a purposive sample of 48 women with physician-diagnosed FM selected from volunteers who agreed to be contacted from a questionnaire study of 176 women with FM; mean age 49 years, divided into 19 women working full- or part-time & 20 women who had stopped working because of FM (had left the labour market or were on 100% long-term sickness benefits or sickness pension) (100% women)	- 45-60 minute individual unstructured audiotaped interviews; interviewers had an interview guide with themes to cover during the interview that could be used as a checklist at the interview end to ensure all themes had been covered<br>- Verbatim transcripts were verified by research team, coded and categorized according to subjects/sub-themes and analysed using NUD*IST software<br>- Participants did not validate interview transcripts.	Medium
Östlund et al., 2004 [96]	To explore lay descriptions of the private arena in relation to MSD. What aspects of the private arena hindered or promoted rehabilitation? How did the interviewees, both men and women, handle household and family duties in relation to decreased functional and work capacity? To expand our understanding of how to empirically study gender relations in qualitative studies comprising both men and women.	20 Swedish workers, most on part-time disability, interviewed in 1997 or 1998, most of whom were blue-collar workers married/cohabiting with children, who had been drawn strategically, based on sex & level of sick leave on a 1995 questionnaire, from a cohort of individuals identified in 1985, 25-34 years old newly sick-listed ≥28 days for neck, shoulder or back MSD living in the same Swedish community (50% women)	- 1-3-hour interviews on what had hindered or promoted rehabilitation & on what the interviewee considered important in the rehabilitation process<br>- 3 core domains emerged, i.e. the private, occupational & health care arena; only the private arena is analyzed in this paper.<br>- Men & women's statements regarding the private arena were compared.<br>- Transcripts were validated by interviewers (not participants).<br>- Authors presented their results to external researchers to avoid any gender bias given that they are a female research group (reflexivity).	Medium

Study	Study objective(s)	Participants analyzed	Study design/method	Study quality
Kvam et al., 2013 [95]	To explore how participation is perceived in important domains of life by men and women with chronic musculoskeletal pain undergoing vocational rehabilitation. 2 specific research questions: (i) How is participation and the process of change in participation in work, family & leisure activities perceived by men & women with chronic musculoskeletal pain undergoing vocational rehabilitation? and (ii) How may the experiences of participation & change in participation in work, family and leisure activities for men & women with chronic musculoskeletal pain affect the process of vocational rehabilitation?	10 Norwegian outpatients 26 to 57 years old with prolonged musculoskeletal pain (back, shoulder or neck pain, FM, arthritis, rheumatism) as a main reason for reduced function & ability to work, undergoing a multidisciplinary vocational rehabilitation program 3 days/week. Outpatients volunteered to take part in the study (purposive sample). Gender samples are balanced for number of children, years with musculoskeletal pain & marital status (female sample includes a greater number of younger participants) (no information on socioeconomic status, although authors state that all participants were economically secure, no information on participants' occupations or physical or psychosocial work demands) (60% women)	- 30-120-minute semi-structured in-depth interviews based on 4 main themes: participation in work, in family, in vocational rehabilitation & general comprehension of participation (in later interviews, leisure activities added)<br>- Gender analysis was carried out in a later stage, once the following 3 categories/trajectories of participation had emerged: "participating as before", "participation constantly changing" & "work on hold".<br>- Participants did not validate the data/themes.	Medium
